# Plastid genome and composition analysis of two medical ferns: *Dryopteris crassirhizoma* Nakai and *Osmunda japonica* Thunb.

**DOI:** 10.1186/s13020-019-0230-4

**Published:** 2019-03-14

**Authors:** Liang Xu, Yanping Xing, Bing Wang, Chunsheng Liu, Wenquan Wang, Tingguo Kang

**Affiliations:** 1 School of Chinese Materia Medica, Beijing University of Chinese Medicine, Beijing, China; 2 School of Pharmacy, Liaoning University of Traditional Chinese Medicine, Dalian, China; 3 Institute of Medicinal Plant Development, Beijing, China

**Keywords:** *Dryopteris crassirhizoma* Nakai, *Osmunda japonica* Thunb., Plastid genome, Composition analysis

## Abstract

**Background:**

*Dryopteris crassirhizoma* Nakai and *Osmunda japonica* Thunb. are ferns that are popularly used for medicine, as recorded by the Chinese pharmacopoeia, and are distributed in different regions of China. However, *O. japonica* is not record in the Standards of Chinese Herbal Medicines in Hong Kong. Research on identification methods of *D. crassirhizoma* and *O. japonica* is necessary and the phylogenetic position of the two species should be identified. The plastid genome is structurally highly conserved, providing valuable sources of genetic markers for phylogenetic analyses and development of molecule makers for identification.

**Methods:**

The plastid genome DNA was extracted from both fern species and then sequenced on the Illumina Hiseq 4000. Sequences were assembled into contigs by SOAPdenovo2.04, aligned to the reference genome using BLAST, and then manually corrected. Genome annotation was performed by the online DOGMA tool. General characteristics of the plastid genomes of the two species were analyzed and compared with closely related species. Additionally, phylogenetical trees were reconstructed by maximum likelihood methods. The content of dryocrassin of the two species were determined according to the Standards of Chinese Herbal Medicines in Hong Kong.

**Results:**

The genome structures of *D. crassirhizoma* and *O. japonica* have different characteristics including the genome size, the size of each area, gene location, and types. Moreover, the (simple sequence repeats) SSRs of the plastid genomes were more similar to other species in the same genera. Compared with *D. fragrans*, *D. crassirhizoma* shows an inversion (approximately 1.6 kb), and *O. japonica* shows two inversions (1.9 kb and 216 bp). The nucleotide diversity (polymorphism information, Pi) analysis showed that the *psbK* gene and *rpl14*-*rpl16* region have the highest Pi value in *Dryopteris*, and the *ycf2*-CDS3 and *rpl14*-*rpl16* regions show the highest Pi vale in *O. japonica*. Phylogenetic analyses showed that the two species were grouped in two separate clades from each other, with both individually located with other members of their genus. The marker content of dryocrassin is not found in *O. japonica*.

**Conclusions:**

The study is the first to identify plastid genome features of *D. crassirhizoma* and *O. japonica*. The results may provide a theoretical basis for the identification and the application of the two medically important fern species.

**Electronic supplementary material:**

The online version of this article (10.1186/s13020-019-0230-4) contains supplementary material, which is available to authorized users.

## Background

The chloroplast is the key organelle for photosynthesis and carbon fixation; chloroplasts also play vital roles in other aspects of plant physiology and development, including the synthesis of amino acids, nucleotides, fatty acids, phytohormones, vitamins, metabolites, and the assimilation of sulfur and nitrogen [1, 2]. In general, plastid genomes are structurally highly conserved across land plants, thus are valuable sources of genetic markers for phylogenetic analyses because of their very low level of recombination [3–5]. Some regions can also be used as DNA barcodes, such as *psbH* and *rbcL*, which provide the necessary universality and species discrimination [6]. Nguyen et al. developed many authentication markers for five major *Panax* species via comparative analysis of complete plastid genome sequences [7]. *Aconitum coreanum* (Levl.) Rapaics also contains a barcoding target sequence in a divergent region, *ndhC*–*trnV*, and a sequence characterized amplified region (SCAR) marker was successfully developed for its discrimination [8]. Comparative plastid genomic studies also provide an invaluable source of information for understanding plant evolution and phylogenetics [9]. Therefore, many studies on the plastid genome from different species have been reported. These studies have shown that the plastid genome gene content and orientation are considerably conserved between species [10]; however, there are also some changes such as in the size, gene intron gains and losses, expansion/contraction of inverted repeats (IRs), structure rearrangements, and inversions [11, 12]. A previous report suggested that the complete plastid genome sequence of ferns has undergone two major rearrangements, distinguishing higher leptosporangiate ferns from basal fern lineages [3]. The IRs of Polypodiales plastomes are dynamic, driven by such events as gene loss, duplication, and putative lateral transfer from mitochondria [13]. The fern also shows the major reduction in the rate of evolution, and there has been a major slowdown in the rate of mutation in *Dicksonia squarrosa* and *Tmesipteris elongata* [14]. Ferns also hold a critical phylogenetic position as the extant sister group to seed plants [15]. Therefore, understanding the organization and structure of fern plastid genomes provides useful information for the studies of ferns.

For Chinese herbal medicines plastid genes, such as *psbA*-*trnH*, *rbcL*, and *matK*, are usually used as DNA barcodes for plants [16]. In addition, *trnL*-*F* has also been used to study the phylogeography and demographic history of *Chrysanthemum indicum* L. [17]. However, DNA barcodes have limited resolution at the species level. The plastid genome of the common Chinese medicine *Panax notoginseng* (Burk.) F.H. Chen from different producing areas sometimes shows differences in length. Specifically, in Wenshan, Yunnan Province, the plastid genome size is 156,466 bp (Genbank Number: KJ566590) and in Wuliang Mountain, Yunnan Province, the plastid genome size is 156,324 bp (Genbank Number: KT001509). However, there is little structural difference between the two samples [18, 19]. This also occurs in *C. indicum* from difference regions (Genbank Number: JN867592, NC_020320).

Ferns are a large group of vascular plants with approximately 2129 species in China [20]. The family includes many medicinally important species such as *Dryopteris crassirhizoma* Nakai and *Osmunda japonica* Thunb.. The roots of these species are widely used in Traditional Chinese Medicine with the effect of clearing heat, detoxifying, and deworming, and the effects are recorded in the Chinese pharmacopoeia [21]. *D.* *crassirhizoma* is distributed in northeast and northern China, and *O. japonica* is the most common species of ferns in warm temperate and subtropical regions of China [22, 23]; its young leaves are rich in nutrients and edible as a wild vegetable. The two species have the same effect described in the Chinese pharmacopoeia; however, there is a difference in the chemical composition of the two species. Dryocrassine is the index component of *D. crassirhizoma* [24], and osmundacetone is the specialty component of *O. japonica* [25]. The species are also used in different prescriptions. Specifically, *O. japonica* is not record in the Standards of Chinese Herbal Medicines in Hong Kong [24]. Previous studies have reported the identification of *D. crassirhizomatis* based on plastid barcodes using *psbA*-*trnH.* This barcode can identify *D. crassirhizomati*s and its adulterants [26]. In addition, *psbA*-*trnH* and *rbcL* can distinguish *O. japonica* from its adulterants [27]. The *O. japonica* plastid genome regions, including *rbcL*, *accD* genes for ribulose 1,5-bisphosphate carboxylase/oxygenase, acetyl-CoA carboxylase beta subunit, and partial cds, have been reported (GenBank: AB494712.1). Even though studies on the plastid genes of these two species have been reported, their phylogenetic positions are vague and the identification from closely related species based on barcodes has not been reported. A comparative analysis about the content of dryocrassin in the two species is unclear. Here, we determined the dryocrassin components of the two species and sequenced and analyzed the complete plastid genomes of *D. crassirhizomatis* (Dryopteridaceae) and *O. japonica* (Osmundaceae). A comparative analysis was conducted with the closely related species to provide information for the quality control of related medicinals and allow for a better understanding of plastid genome evolution within their respective families.

## Methods

### DNA extraction and sequencing

We collected leaves from one individual of *D.* *crassirhizoma* and *O. japonica*, respectively, from Qianshan, Anshan City, Liaoning Province (N40°39′47.42″, E124°52′13.16″) and Sanming, Jianning City, Fujian Province (N27°49′8.06″, E117°43′10.93″) China. The two species were identified by Kang Tingguo, a professor from Liaoning University of Traditional Chinese Medicine. Voucher specimens were deposited in the Liaoning University of Traditional Chinese Medicine Herbarium (*D.* *crassirhizoma* 20170827001LY, *O. japonica* 20170917001LY).

Plastid DNA was extracted from approximately 5 g fresh, young leaves from *D. crassirhizoma* and *O. japonica* using a modified cetyl trimethylammonium bromide method [28]. After DNA isolation, 1 μg of purified DNA was fragmented and used to construct short-insert libraries (insert size 430 bp) according to the manufacturer’s instructions (Illumina), then sequenced on an Illumina Hiseq 4000 [29]. Prior to assembly, raw reads were filtered. This filtering step was performed to remove reads with adaptors, reads showing a quality score below 20 (Q < 20), reads containing a percentage of uncalled based (“N” characters) equal or greater than 10%, and the duplicated sequences. The plastid genome was reconstructed using a combination of de novo and reference-guided assemblies, and the following three steps were used to assemble plastid genomes [30]. First, the filtered reads were assembled into contigs using SOAPdenovo2.04 [31]. Second, contigs were aligned to the reference genome of two species using BLAST, and aligned contigs (≥ 80% similarity and query coverage) were ordered according to the reference genome. Third, clean reads were mapped to the assembled draft plastid genome to correct any wrong bases, and the majority of gaps were filled through local assembly.

### Genome assembly and annotation

The plastid genes were annotated using the online DOGMA tool (http://dogma.ccbb.utexas.edu/index.html) [32], using default parameters to predict protein-coding genes, transfer RNA (tRNA) genes, and ribosome RNA (rRNA) genes. A whole plastid genome BLAST [33] search (E-value ≤ 1e−5, minimal alignment length percentage ≥ 40%) was performed against five databases: KEGG (Kyoto Encyclopedia of Genes and Genomes) [34–36], COG (Clusters of Orthologous Groups) [37, 38], NR (Non-Redundant Protein Database databases), Swiss-Prot [39], and GO (Gene Ontology) [40]. Sequencing data and gene annotation were then submitted to GenBank and assigned accession number (*D. crassirhizoma*: MK554795, *O. japonica*: MK554796).

### Plastid genome mapping

The plastid genomes of *D.* *crassirhizoma* and *O. japonica* were exported in GenBank format and the plastid genome was mapped using Organellar Genome Draw (OGDRAW) (Max Planck Institute of Molecular Plant Physiology, Am Mühlenberg, Potsdam, Germany) (http://ogdraw.mpimp-golm. mpg.de/index. Shtml) [41].

### Comparative analysis of genomes

The simple sequence repeat (SSR) software MicroSAtellite (MISA) (http://pgrc.ipk-gatersleben.de/misa/) was used to identify the SSR sequences, and tandem repeats of 1–6 nucleotides were considered microsatellites. The minimum number of repeats were set to 8, 5, 4, 3, 3, and 3 for mono-, di-, tri-, tetra-, penta-, and hexa-nucleotides, respectively. The data was compared with *Osmundastrum cinnamomeum* L. (*O. cinnamomea* L.) (NC_024157.1), *D. fragrans* (L.) Schott (KX418656), and *D. decipiens* (Hook.) O. Ktze. (NC_035854.1). We focused on perfect repeat sequences [42]. Long repeat sequences of the two species were identified using REPuter. Four types of repeats (dispersed, tandem, palindromic, and gene similarity repeats) were determined. The maximal length of the gap size between palindromic repeats was 3 kb. Overlapping repeats were incorporated into one repeat motif whenever possible and a given region in the genome was defined as having only one type of repeat. When one repeat motif could be described as both tandem and dispersed, the region was described as a tandem repeat rather than a dispersed repeat [43]. The alignments were visually checked and edited manually. The gene order of the plastid genomes of *D. crassirhizoma* and *O. japonica* were compared with *D. fragrans* using online zPicture software (http://zpicture.dcode.org/) [44]. To screen variable characters between *D.* *crassirhizoma,* *D. fragrans*, and *D. decipiens* and between *O. japonica* and *O. cinnamomeum,* the average number of nucleotide differences and total number of mutations were determined to analyze nucleotide diversity (polymorphism information, Pi) using DnaSP v5.0 [45].

### Phylogenetic analysis

To identify the phylogenetic position of *D.* *crassirhizoma* and *O. japonica* and their relationship with other families, phylogenetic trees were constructed by the plastid genome sequences from 30 species, and 28 species were download from GenBank. Among them, two species, *Selaginella uncinata* (Desv.) Spring and *Selaginella moellendorffii* Hieron., were set as outgroups. The analysis was run using the whole plastid genomes single nucleotide polymorphisms (SNPs). A Maximum Likelihood (ML) phylogenetic tree was constructed using PhyML 3.0, and the model GTR + I + G was selected for the ML analyses with 100 bootstrap replicates to calculate the bootstrap values [46].

### Comparative analysis of dryocrassin content in *D. crassirhizoma* and *O. japonica*

This research used high-performance liquid chromatographic detection the dryocrassin content in *D. crassirhizoma* and *O. japonica* [24]. The Additional file 1: Minimum Standards of Reporting Checklist includes details of the experimental design, statistics, and resources used in this study.

## Results

### Plastid genome structure

**Fig. 1:**
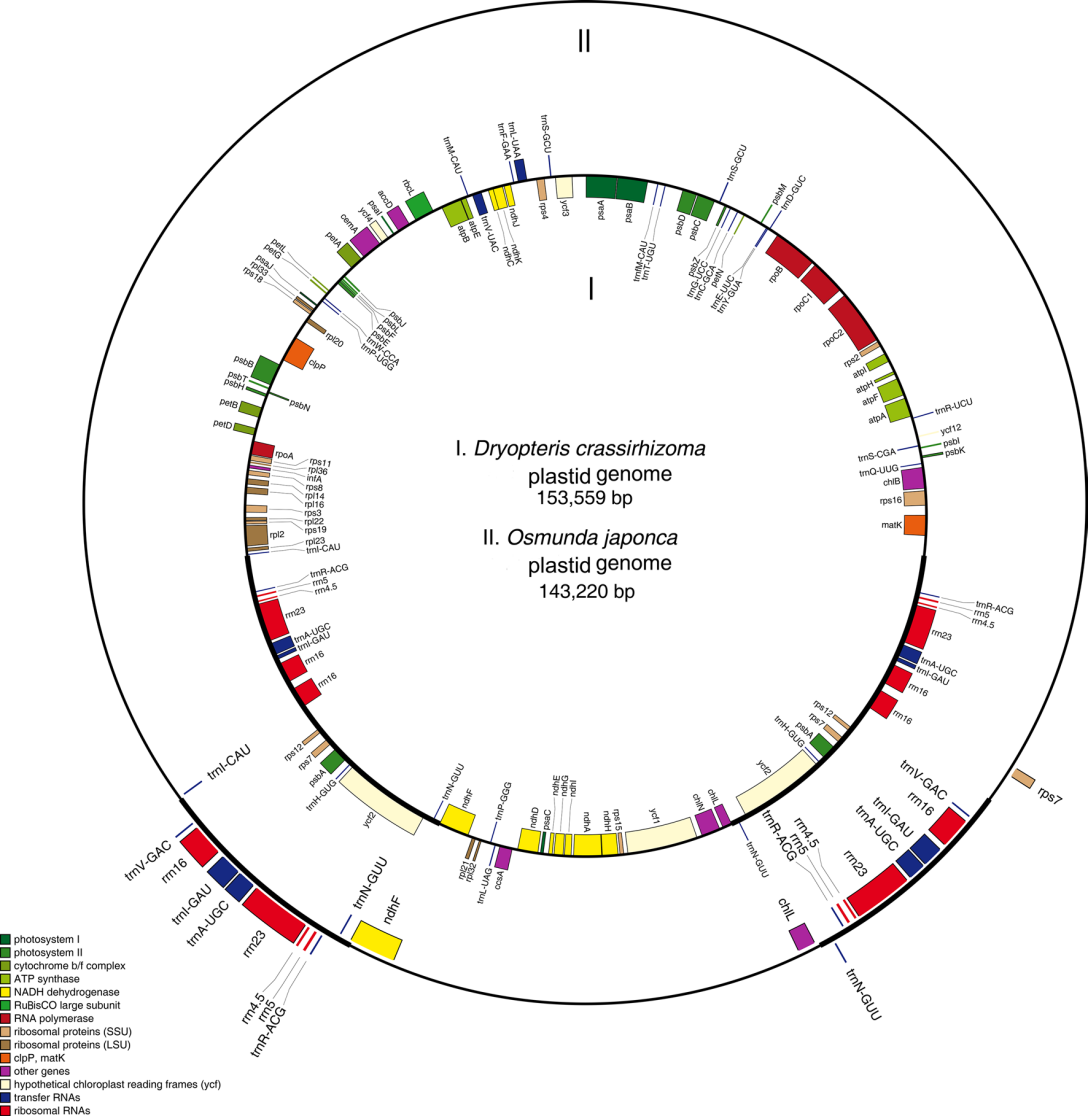
Plastid genome map for two sequenced ferns. Boxes on the inside (I) and outside of the outer circle (II) represent genes of *D. crassirhizoma* and *O. japonica*, respectively

In the fully assembled plastid genome genome sequence from *D. crassirhizoma* and *O. japonica*, the genome size was 153,559 bp and 143,220 bp, the large single copy region (LSC) was 82,495 bp and 100,464 bp, the small single copy region (SSC) was 21,599 bp and 22,224 bp, the IRs were 49,464 bp and 20,532 bp, respectively (Table 1). The plastid genome structure of the two species showed differences in size of the same region (Fig. 1). There were 86 and 84 protein coding genes annotated for *D.* *crassirhizoma* and *O. japonica*, respectively. There were significant differences in length and genes of the IR region between the genome sequences (Fig. 1).

**Table 1:** General features of the plastid genomes in *D.* *crassirhizoma* and *O. japonica*

Species	*D. crassirhizoma*	*O. japonca*
Genome size (bp)	153,559	143,220
GC content (%)	43.39	40.42
LSC length (bp)	82,495	100,464
SSC length (bp)	21,599	22,224
IR length (bp)	49,464	20,532
Protein coding gene number	86	84
tRNA number	31	36
rRNA number	10	8

*D.* *crassirhizoma* contains 31 tRNA genes, 10 rRNA (Additional file 2: Table S1), and 12 tRNA genes, and all rRNAs were located in the IR region (Additional file 2: Table S1). *O. japonica* contains 36 tRNA genes, eight rRNA genes, and 14 tRNAs in the IR region. The number of tRNAs in the two species were different in that *D.* *crassirhizoma* had five fewer tRNAs than *O. japonica.* The gene *trnS*-*CGA* had two copies in *O. japonica* and was in the IR region, but in *D.* *crassirhizoma*, only one copy was present. The gene *trnH*-*GUG* showed an opposite phenomenon. The genes *trnK*-*UUU, trnL*-*CAA, trnR*-*CCG, trnT*-*GGU,* and *trnV*-*GAC* were not present in *D.* *crassirhizoma* and the genes *trnT*-*GGU* and *trnV*-*GAC* were located in the IR region. The genes *trnT*-*UGU* and *trnL*-*UAA* were not present in *O. japonica.* The protein coding gene *ndhB*, *psaM*, and *rps14* were only annotated in *O. japonica* but the gene *ycf1* was absent. The genes *ycf2, rps7, psbA*, *rps12*, and *psbA* were located in the IR region in *D. crassirhizoma.*

Among the protein coding genes, *D. crassirhizoma* was found to have 20 genes contain the introns while *O. japonica* have 12 (Table 2). In *D. crassirhizoma*, the gene *ycf2* encoded four introns, the gene *rpoB* encoded three introns, and the genes *clpP* and *ndhF* encoded two introns. In *O. japonica* the genes *clpP, ycf2*, and *ycf3* encoded two introns.

**Table 2:** Length of intron-containing genes within the *D.* *crassirhizoma* and *O. japonica* plastid genome

	Gene	Exon (bp)	Intron (bp)	Exon (bp)	Intron (bp)	Exon (bp)	Intron (bp)	Exon (bp)	Intron (bp)	Exon (bp)
*D. crassirhizoma*	*matK*	1239	9	252						
	*rps16*	213	824	45						
	*chlB*	1161	135	249						
	*atpA*	495	3	900						
	*atpF*	435	704	147						
	*rpoC1*	1642	674	257						
	*rpoB*	633	153	672	75	927	39	423		
	*ycf3*	340	758	146						
	*petA*	276	12	684						
	*clpP*	281	534	292	691	66				
	*rps11*	270	2	120						
	*infA*	120	57	66						
	*rpl2*	375	780	354						
	*rps7*-*D2*	138	3	312						
	*ycf2*-*D2*	195	3	1046	216	2054	3	894	6	1734
	*ndhF*	1545	54	228	48	354				
	*ndhA*	581	919	559						
	*ycf1*	4125	21	993						
	*ycf2*	1734	6	894	3	2055	216	1146	3	195
	*rps7*	312	3	138						
*O. japonica*	*atpF*	411	644	158						
	*clpP*	255	533	291	704	69				
	*ndhA*	552	873	558						
	*ndhB*	723	720	762						
	*petB*	6	759	642						
	*rpl16*	429	752	9						
	*rpl2*	452	665	397						
	*rpoC1*	1626	641	432						
	*rps12*	315	82,982	114						
	*rps16*	213	770	42						
	*ycf2*	5491	229	23	5	1584				
	*ycf3*	206	620	33	691	124				

### Repeat sequences analysis

#### SSR sequence analysis

**Fig. 2:**
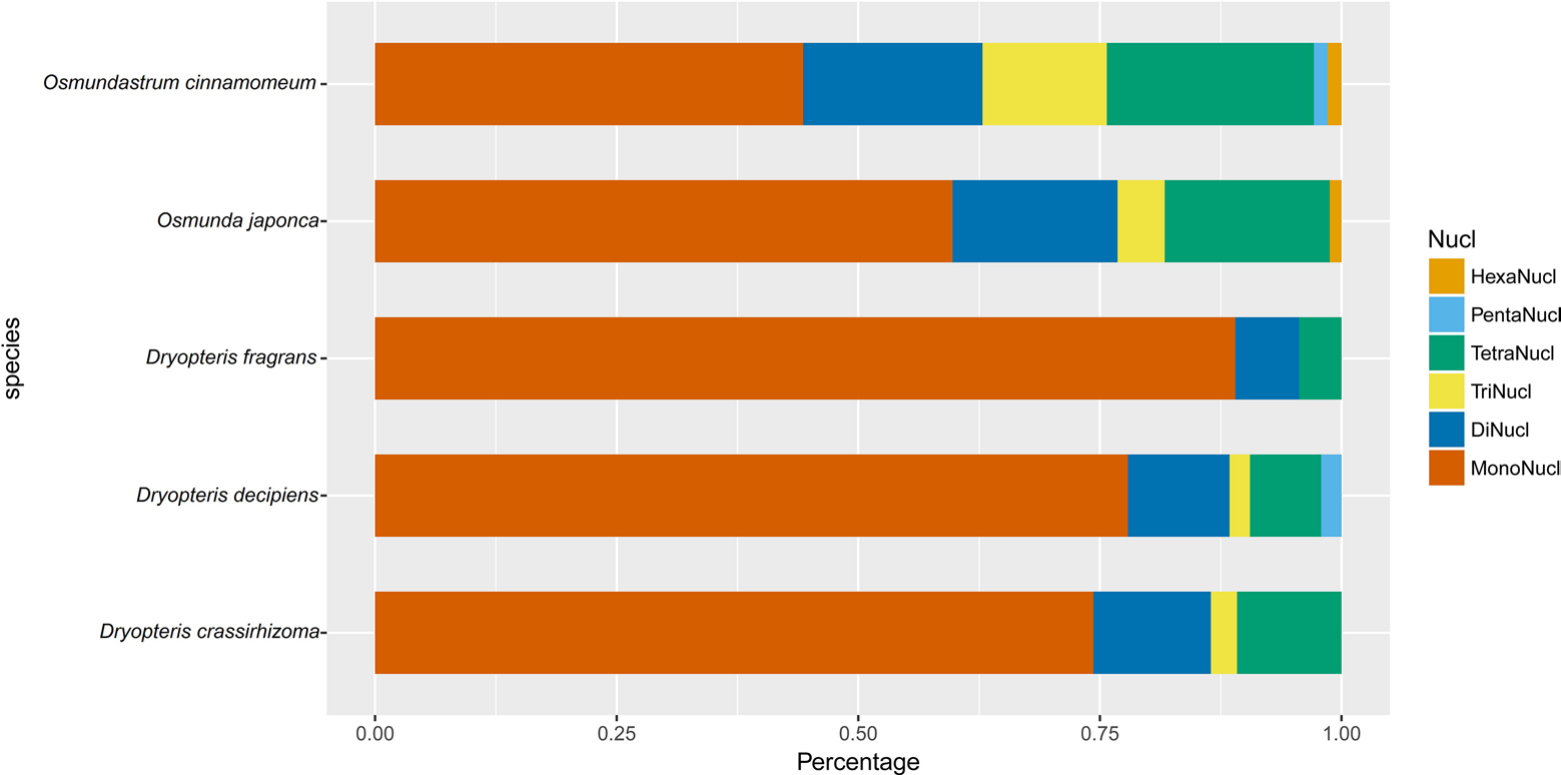
SSRs in the plastid genomes of *O. japonica*, *D. crassirhizoma*, and three other fern species. Mono represents mononucleotide repeats, Di represents dinucleotide repeats, and Tri represents trinucleotide repeats

A total of 74 and 82 SSR locis that were 1024 bp and 1191 bp long, respectively, were detected in the *D.* *crassirhizoma* and *O. japonica* plastid genomes (Table 3). The number of mono-repeats were dominant in the plastid genomes of both species. There were 54 and 62 SSRs located in the LSC, 14 and 12 located in the IR, and six and eight located in the SSC in the *D. crassirhizoma* and *O. japonica* plastid genome, respectively (Additional file 3: Table S2, Additional file 4: Table S3). Compared with *D. fragrans, D. decipiens,* and *O. cinnamomeum* (Fig. 2), we found that the three *s*pecies from *Dryopteris* (Dryopteridaceae) had more SSR mono-repeats than the two species from *Osmunda*.

**Table 3:** SSRs in *O. japonica* and *D.* *crassirhizoma* plastid genome

Unit size	*O. japonica*	*D. crassirhizoma*
	Number of SSRs	Number of SSRs
MonoNucl	49	55
DiNucl	14	9
TriNucl	4	2
TetraNucl	14	8
PentaNucl	0	0
HexaNucl	1	0

### Large repeat analysis

Large repeat sequences showed repeats with lengths of ≥ 30 bp each. Twenty-six and 111 pairs of large repeat sequences with sequence identity of > 90% were found in the *O. japonica* (Additional file 5: Table S4) and *D.* *crassirhizoma* (Additional file 6: Table S5) plastid genomes. The repeats from *D.* *crassirhizoma* ranged from 30 to 145 bp in length, and in *O. japonica*, the repeats ranged from 30 to 46 bp in length. A total of 10 and 87 large repeat sequences were located in the genes in *O. japonica* and *D.* *crassirhizoma*, respectively.

### Sequence order

**Fig. 3:**
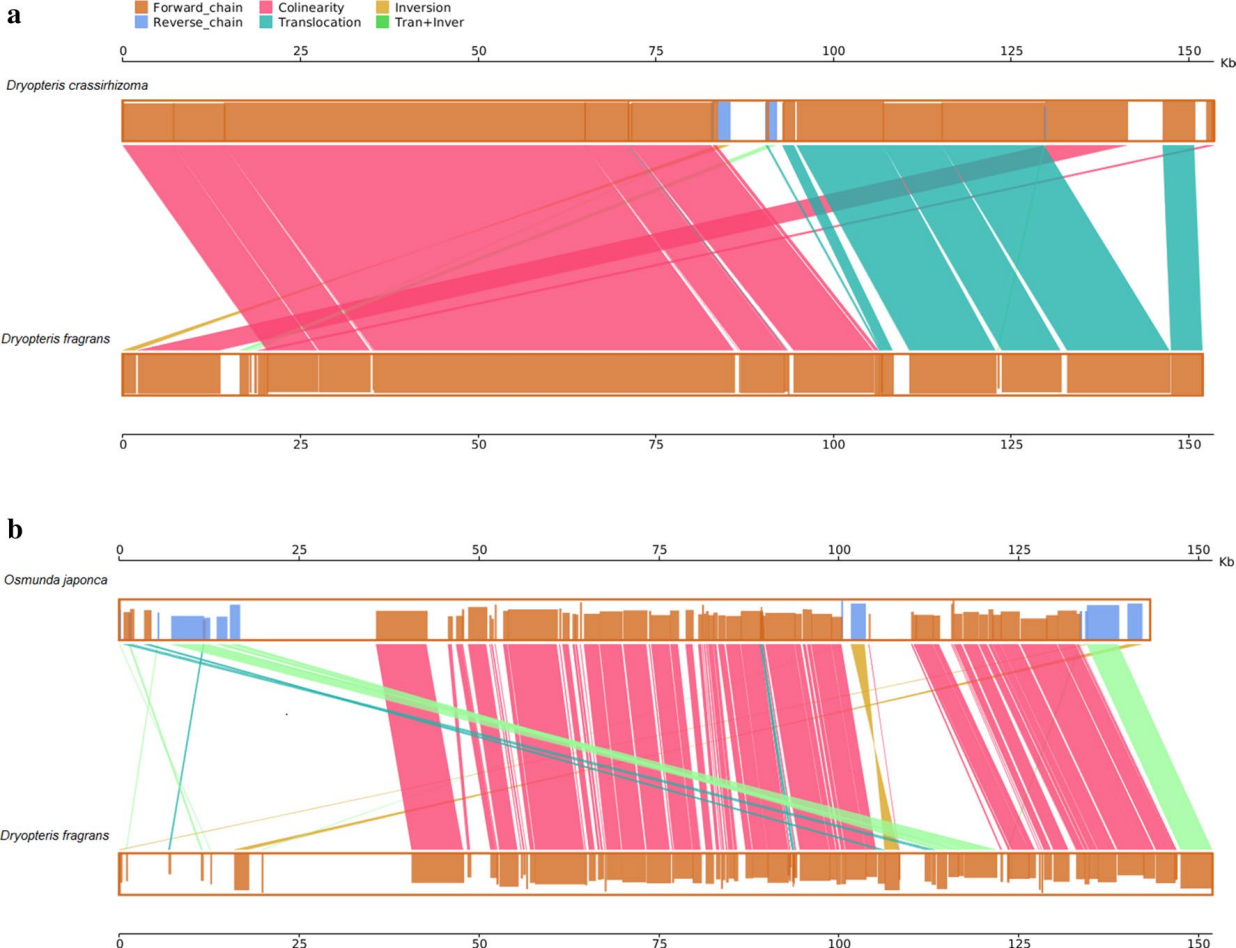
Comparisons of the gene order of the *D. crassirhizoma* (**a**) and *O. japonica* (**b**) plastid genome with the gene order of the plastid genome of *D.* *fragrans*

Compared with *D.* *fragrans,* the *D.* *crassirhizoma* plastid genome showed a high degree of collinearity and translocation. Furthermore, it also showed low degree of transinversion (Fig. 3a). The *D.* *crassirhizoma* plastid genome shares one approximately 1.6 kb long inversion with the *D.* *fragrans* plastid genome. The inversion is located at the beginning of the IR between the *trnS*-*CGA* and *ycf12*. Compared to the *D.* *fragrans* plastid genome, the *O. japonica* plastid genome showed a high degree of collinearity (Fig. 3b). It also showed a low degree of transinversions. Compared with *D. fragrans, O. japonica* showed two inversions, with one approximately 1.9 kb long at the beginning of LSC and the other about 216 bp long located in SSC.

### LSC, SSC, and IR border regions analysis

**Fig. 4:**
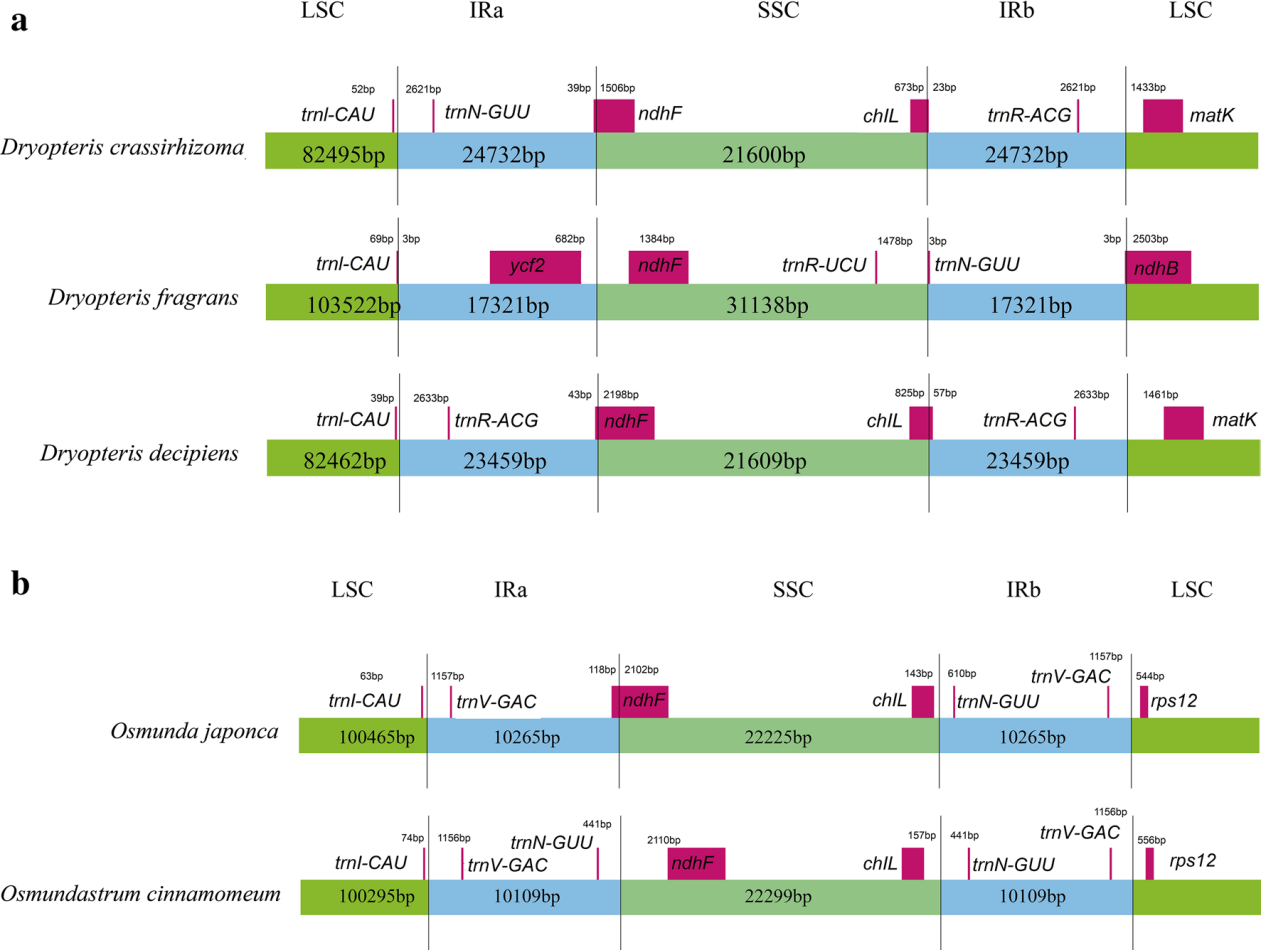
Comparisons of LSC, SSC, and IR border regions among three *Dryopteris* species (**a**) and two *Osmunda* (**b**) species

We analyzed the border structures of three *Dryopteris* species (Fig. 4a) and two *Osmunda* species (Fig. 4b). The lengths of the IR regions of the *Dryopteris* species were different and ranged from 17,321 to 24,732 bp, and the expansion and contraction of IR regions differed. There were 2673 bp, 72 bp, and 2673 bp no-coding regions in *D. crassirhizoma*, *D. fragrans*, and *D. decipiens* in the IRa/SSC boundary, respectively. The *ndhF* gene of *D. fragrans* was located in the LSC regions; the *ndhF* gene of the other two species extended into the IRa regions. The *chlL* gene was located in the SSC and extended into the IRb in *D. crassirhizoma* and *D. decipiens*. However, in *D. fragrans* there was no gene here. The gene *ndhB* located in the LSC and extended into IRb in *D. fragrans*. In *D. crassirhizoma* and *D. decipiens* there was no gene at the IRb/LSC border. The adjacent genes were *matK*.

The boundary characteristics of *O. japonica* and *O. cinnamomeum* were similar. The lengths of the IR regions of *O. japonica* and *O. cinnamomeum* were 10,265 bp and 10,109 bp. The *ndhF* genes of *O. japonica* located in the SSC region and extended into the IRa region and were 118 bp long. In *O. cinnamomeum*, there was a no-coding region (2110 bp) adjacent the IRa/SSC border.

### Nucleotide diversity analysis

**Fig. 5:**
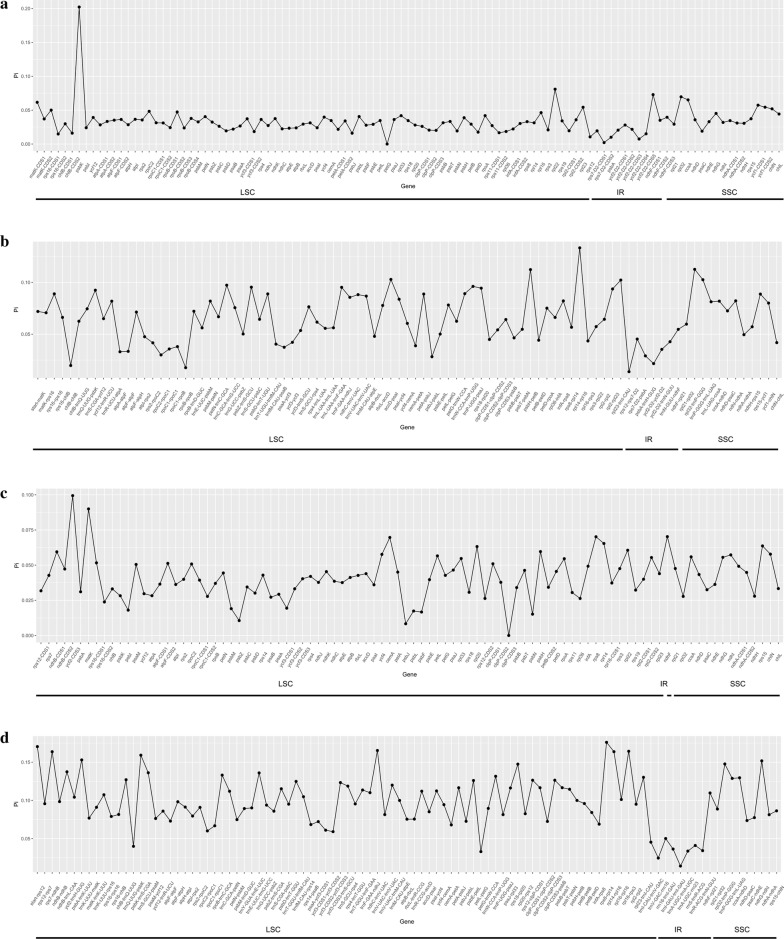
Comparative analysis of the nucleotide variability by Pi values within *Dryopteris* (**a** coding region, **b** non-coding region) and within *Osmunda* (**c** coding region, **d** non-coding region)

Plastid genome sequences contain regions that are highly variable. Such regions (coding and non-coding regions) are useful for the screening of suitable loci to resolve closely related species or genera in phylogenetic analyses and for DNA barcoding. The coding genes and non-coding regions of three *Dryopteris* species and two *Osmunda* species were compared (*D. crassirhizoma* was compared with *D. fragrans* and *D. decipiens*, and *O. japonica* was compared with *O. cinnamomeum*). We generated 107 coding genes (Fig. 5a) and 91 non-coding genes (Fig. 5b) within *Dryopteris*. Among the Pi values obtained from the comparative analysis (Additional file 7: Table S6), we found Pi values were ranged from 0.0000 (*petG* gene) to 0.20222 (*psbK* gene) of the coding genes, and most Pi values of the gene were greater than 0.01. The Pi value of non-coding genes loci ranged from 0.01380 (*rps12*-*rps7*-D2 region) to 0.13333 (*rpl14*-*rpl16* region) of the non-coding genes loci. The coding gene regions were much more conserved than the non-coding gene regions. The first two significant variable loci were the gene *psbK* (Pi = 0.20222) and the *rpl14*-*rpl16* region (Pi = 0.13333), and they both were located in the LSC region.

Within *Osmunda*, we generated 94 coding genes (Fig. 5c) and 101 non-coding genes (Fig. 5d). The Pi values ranged from 0.0000 (*clpP*-CDS3 gene) to 0.09942 (*ycf2*-CDS3 gene) of the coding genes and most Pi values of the genes were greater than 0.01 (Additional file 8: Table S7). Pi values were ranged from 0.01380 (*rps12*-*rps7*-D2 region) to 0.13333 (*rpl14*-*rpl16* region) of the non-coding genes. The significant variable loci (*ycf2*-CDS3 gene and *rps12*-*rps7*-D2 region) were located in the LSC region.

### Phylogenetic analysis

**Fig. 6:**
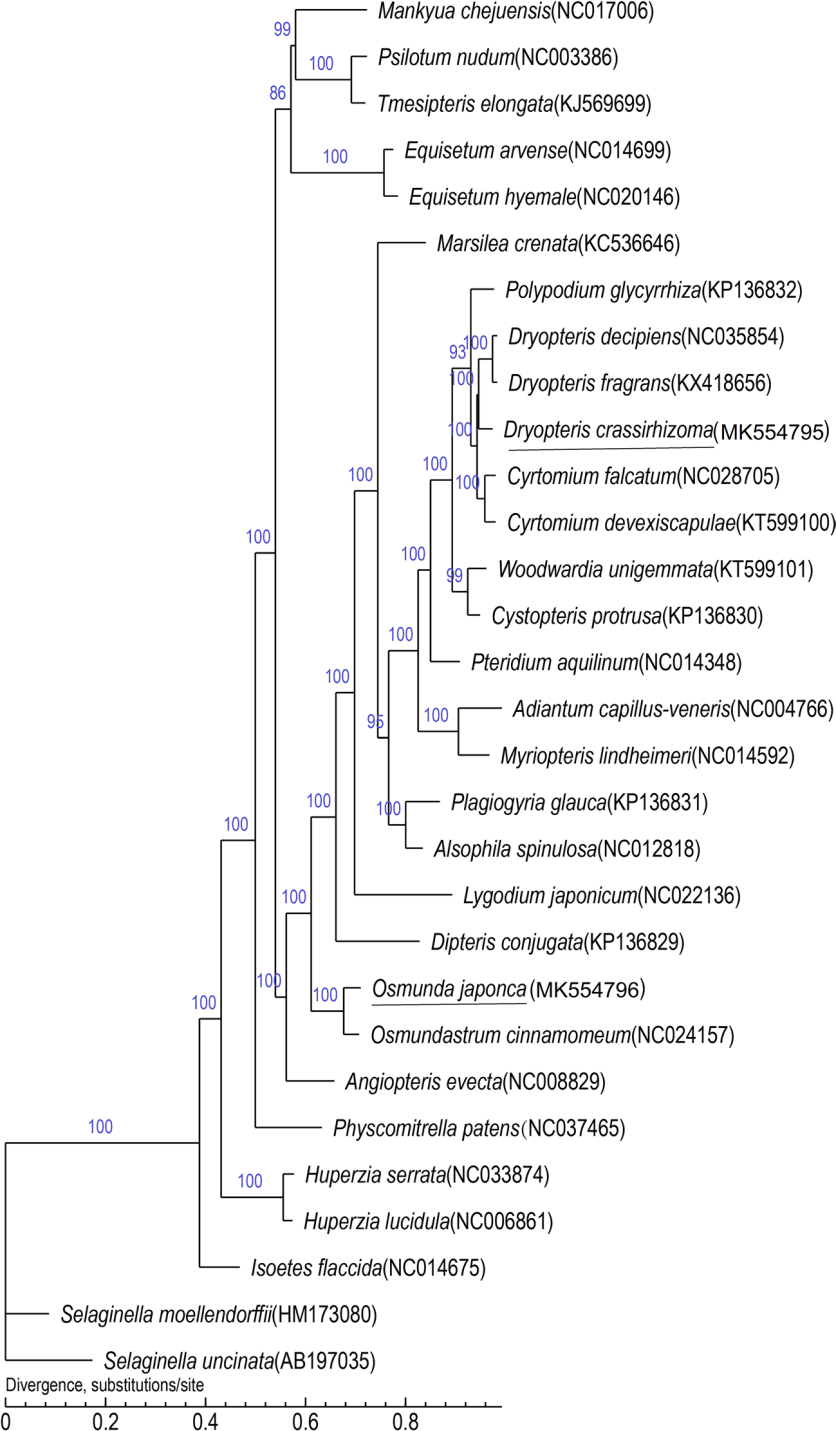
Phylogenetic relationships of 30 species. The ML method used 1000 reiterations for the bootstrapping analysis

In this study, we investigated the phylogenetic relatedness among the plastid genomes of 30 species. There were 22 nodes with support values of 100% and four nodes with support values greater than 90%. The ferns of Leptosporangiatidae, Psilophytinae, and Equisetinae were grouped into three separate clades, respectively. The two Eusporangiate ferns were not grouped in one clade, with *Mankyua chejuensis* B.Y. Sun was closer to Psilophytinae and the other one *Angiopteris evecta* (G. Forst.) Hoffm. (Marattiaceae) was identified as a sister genus to Leptosporangiatidae. The two ferns we study were showed 100% homology with ferns from their respective families in the phylogenetic trees. The Leptosporangiatidae ferns formed two clades: *Osmuda* and the other clade contained the other species. This tree also indicated that the moss *Physcomitrella patens* (Hedw.) Bruch & Schimp was grouped in one clade with Leptosporangiatidae, Psilophytinae, Equisetinae, and Eusporangiate (Fig. 6).

### Comparative analysis of dryocrassin content in *D.* *crassirhizoma* and *O. japonica*

We were able to determine the content dryocrassin in *D.* *crassirhizoma.* The content has the same relative retention time as the marker content dryocrassin when detected by HPLC, but dryocrassin was not detected from the *O. japonica*.

## Discussion

### Plastid genome structure

Searching the whole plastid genome to identify the most variable regions and focusing on regions for mini-barcodes are believed to be efficient methods for developing taxon-specific DNA mini-barcodes [18]. And the identification of a medicinal material at the DNA level provides an objective and powerful tool for quality control [47]. For Chinese herbal medicine, there is insufficient evidence if Chinese herbal medicines from different producing areas would show differences in the plastid genome. The plastid genome of the same species from different regions does not show significant differences, but we could see that variation still exists [18, 19]. And the universal conventional DNA barcodes developed from the plastid genomes can rarely be used for identification within species. But, for many plant species, comparing the plastid genome with related species shows relatively conservative gene content [48] and also can be identified by the universal conventional DNA barcodes developed from the plastid genomes [7, 8]. We thought studying the plastid genome may significant in studying the origin traceability of Traditional Chinese Medicine.

The total size of the *D.* *crassirhizoma* plastid genome was slightly little difference from the plastid genome of another species within the same genus (*D.* *fragrans,* KX418656, 151,978 bp and *D.* *decipiens,* NC_035854.1, 150,987 bp) [49]. This is similar to *Amomum compactum* Soland ex Maton, which has a complete plastid genome is different from slightly from the other close relative species [50]. The total size of the *O. japonica* genome was larger than the genome size of *Osmunda* species (*O. cinnamomeum,* NC_024157.1, 142,812 bp) [9]. Such differences in plastid genome size may result from the expansion and contraction of the border areas between IR regions and single copy regions [51]. The related ferns had a relatively conservative genome size that was similar to many Angiosperms [8, 48].

Compared to *O. japonica* the tRNA genes *trnK*-*UUU, trnL*-*CAA, trnR*-*CCG, trnT*-*GGU,* and *trnV*-*GAC* were not present in *D. crassirhizoma.* All of these tRNA genes, except *trnT*-*GGU*, were also not present in the *Cyrtomium falcatum* (L.f.) Presl [51]. However, *trnI*-*GAU* was present in *D.* *crassirhizoma* and *C. falcatum*, but it was not present in many other *Dryopteris* species [13]. The *trnR*-*CCG* was present in *O. japonica*, which was consistent with previous studies that detected intact one in Gleicheniales, Hymenophyllales, and Osmundales [3, 52]. The genes *trnT*-*UGU* and *trnL*-*UAA* were not present in *O. japonica*. The *trnT*-*UGU* was also detected in *Alsophila spinulosa* (Wall. ex Hook.) R. M. Tryon [3]. The protein gene *psaM* was present in *O. japonica* but not in *D. crassirhizoma*. This gene also been detected in *Psilotum* [53] and *Angiopteris* [54], but not in *Adiantum* [55], suggesting that variation in genes is common.

The introns in eukaryotes can be applied to study phylogenetic evolution, evolutionary distance, and the regulation of gene expression [56]. Among the protein coding gene genes, *D. crassirhizoma* had more genes contained introns. The two ferns both had the *RpoC1* intron, but many ferns of the genus *Lygodium* have lost the *RpoC1* intron [9]. Those plastid genome modifications, such as gene/intron gains or losses, can be used to describe characters.

### Repeat sequences analysis

Plastid genome SSRs are also the effective molecular markers to study polymorphisms and have been used within sunflower (*Helianthus annuus*) [57]. The results show that *D. crassirhizoma* had more SSRs in its plastid genome than *O. japonica*. The three *Dryopteris* species had more mono-repeat SSRs than the plastid genomes of the two species from *Osmunda*. Within the same family, the species have similar SSR compositions. This may also demonstrate the conservativeness of the plastid genome [3]. We also found numerous of long repeated sequences in *D.* *crassirhizoma* and *O. japonica*. The lengths of the repeats found in *O. japonica* represent much shorter repeats than those in *D. crassirhizoma.* The majority of long repeated sequences were located in noncoding regions that have been reported in several Angiosperm lineages [58]. Our data will contribute to further research on population genetics and phylogeography of these two fern genera.

### Sequence order

Rearrangements of sequence order in fern have been reported before [3]. The two ferns were compared with *D. fragrans*. They all showed a high degree of collinearity and one or two inversions. The *Alsophila* plastid genome also has been reported shares three key inversions with other ferns relative to bryophytes [3]. All the inversions were located at the beginning of the IR, which may because of high variability in the IR sequence and gene content [14]. Inversions may very common within the ferns. This would contribute to the study of kinship of different species.

### LSC, SSC, and IR border regions analysis

Although the IR regions are highly conserved, the expansion and contraction of IR regions is the general feature of plastid genomes, and they are mainly responsible for variations in plastid genome size and rearrangement [59, 60]. *Dryopteris* species have relatively different boundary characteristics with the length and the expansion and contraction of IR regions. This has mainly been reflected in the presence or absence of boundary genes and their size. The boundary characteristics of *O. japonica* and *O. cinnamomeum* were relatively similar; however, the two species we studied show great differences. The plastid genomes in closely related species also exhibit considerable variation in ferns, which was different from that in the Angiosperms [8]. However, these hypotheses need require the testing of more plastid genome sequences in the future.

### Nucleotide diversity analysis

In the plastid, genome mutation events are usually gathered in “hot spots” and these mutational dynamics created highly variable regions dispersed throughout the plastid genome [61, 62]. The highly variable regions, *trnH*-*psbA*, *trnR*-*atpA*, *atpI*-*rps2*, *rps2*-*rpoC2*, *petN*-*psbM*, *rps4*-*trnT*, and *rpl33*-*rps18*, between *Oresitrophe* and *Mukdeni* have been report before [63]. Within *Dryopteris* the IR region was much more conserved than the LSC and SSC regions in both coding and non-coding regions, which was similar to previous studies [63]. Analysis of the five species indicated that the coding region was more conserved than the non-coding region. Non-coding sequences of the plastid genome are a primary source of data for molecular systematics, phylogeographic, and population genetic studies of plants [61], thus this would provide important genetic information for subsequent studies on phylogeography and divergence history of *Dryopteris* and *Osmunda* species.

### Phylogenetic analysis

Plastid genome data are beneficial in resolving species definitions because organelle-based “barcodes” can be established for a species and then used to unmask interspecies phylogenetic relationships [58]. The phylogenetic relationships among many ferns have been studied through different methods, and at the broadest level, our results were congruent with previous studies [64, 65]. The ferns of Psilophytinae formed a sister clade to Equisetinae with strong support, which was different from a previous study [29]. It is thus necessary to expand taxon sampling as the next step in future phylogenomic analyses of polypods to confirm the position of ferns. The two ferns of eusporangiate were not grouped in one clade and *M. chejuensis* was more closely related to Psilophytinae, which was similar to previous reports [14]. The phylogenetic relationships among the two eusporangiate ferns are still uncertain [9]. The position of *Osmuda* may indicate that *Osmuda* diverged early in the lineage of leptosporangiate ferns [9]. The position of the two fern species that we study were consistent with morphological classification, thus the plastid genome may become an important assistant method for species classification.

### Comparative analysis of dryocrassin content in *D.* *crassirhizoma* and *O. japonica*

We can determine the marker content of dryocrassin from *D. crassirhizoma*, but it was not detected from *O. japonica*. Even though they were both recorded in the Chinese Pharmacopoeia to have a similar curative effect [17], their chemical composition is different. It seems that they have different active sites, so the identification is necessary.

## Conclusions

In this study, we conducted plastid genome skimming for *D.* *crassirhizoma* and *O. japonica.* By comparing and analyzing these data, we found the structure of the two plastid genomes was very different and the main active components are also different. Different SSR features may be able to be used to develop molecular markers for molecular identification and genetic diversity. The genomes also show a certain number of inversions and translocations when compared with the other fern species. In addition, the nucleotide diversity provides a reference for studying the genetic variation of the two species. The genomic structure and genetic resources presented in this study contribute to further studies on population genetics, phylogenetics, and conservation biology of ferns.

## Additional files

**Additional file 1.** Minimum standards of reporting checklist.

**Additional file 2: Table S1.** List of genes obtained from the *D.* *crassirhizoma* Nakai and *O. japonca* Thunb. plastid genome sequences.

**Additional file 3: Table S2.** The SSR characteristic in *Dryopteris* *crassirhizoma* Nakai.

**Additional file 4: Table S3.** The SSR characteristic in *Osmunda japonica* Thunb..

**Additional file 5: Table S4.** Long repeat sequences in *Osmunda japonica* Thunb. plastid genome.

**Additional file 6: Table S5.** Long repeat sequences in *Dryopteris* *crassirhizoma* Nakai plastid genome.

**Additional file 7: Table S6.** The Pi value of coding regions and no-coding regions of *Dryopteris.*

**Additional file 8: Table S7.** The Pi value of coding region and no-coding region of *Osmunda.*

## Abbreviations

LSC: large single copy
SSC: small single copy
IR: inverted repeat
tRNA: transfer RNA
rRNA: ribosomal RNA
