# Supplementary material for: Plastid genome and composition analysis of two medical ferns: Dryopteris crassirhizoma Nakai and Osmunda japonica Thunb
Source: Chin Med. 2019 Mar 14;14:9. doi: 10.1186/s13020-019-0230-4 (PMC6417082; doi:10.1186/s13020-019-0230-4)
Supplement: Supplementary file 2 — Additional file 2: Table S1. List of genes obtained from the D. crassirhizoma Nakai and O. japonca Thunb. plastid genome sequences. [file 13020_2019_230_MOESM2_ESM.doc]

**Table S1 List of genes obtained from the *Dryopteris crassirhizoma* Nakai and *Osmunda japonca* Thunb. Plastid Genome**

|  | ***D. crassirhizoma* Nakai** | ***O. japonica* Thunb.** |
| --- | --- | --- |
| Group of genes | Gene Name | Gene Name |
| Ribosomal RNAs | rRNA23^4^ , 16^5^ , 5^4^ , 4.5^4^ | rRNA23^4^ , 16^4^ , 5^4^ , 4.5^4^ |
| Transfer RNAs | *trnQ-UUG,trnS-CGA,trnR-UCU,trnD-GUC,trnY-GUA,trnE-UUC,trnC-GCA,trnG-UCC,trnS-GCU^4^,trnT-UGU,trnfM-CAU,trnL-UAA,trnF-GAA,trnV-UAC,trnM-CAU,trnW-CCA,trnP-UGG,trnI-CAU,trnR-ACG^4^,trnA-UGC^4^,trnI-GAU^4^,trnH-GUG^4^,trnN-GUU^4^ ,trnP-UGG,trnL-UAG,* | *trnL-CAA,trnH-GUG,trnK-UUU,trnQ-UUG,trnR-UCU,trnC-GCA,trnD-GUC,trnY-GUA,trnE-UUC,trnG-UCC,trnS-CGA^4^,trnfM-CAU,trnS-GCU^4^,trnT-GGU^4^,trnF-GAA,trnV-UAC,trnM-CAU,trnR-CCG,trnW-CCA,trnP-UGG,trnI-CAU,trnV-GAC4^4^,trnI-GAU^4^,trnA-UGC^4^,trnR-ACG,trnN-GUU^4^,trnP-GGG,trnL-UAG,trnR-ACG,* |
| Proteins of small ribosomal subunit | *rps11^1^,rps12^4^,rps15,rps16^1^,rps18,rps19,rps2,rps3,rps4,rps7^1,4^,rps8,* | *rps11,rps12^1^,rps14,rps15,rps16^1^,rps18,rps19,rps2,rps3,rps4,rps7,rps8* |
| Proteins of large ribosomal subunit | *rpl14,rpl16,rpl2^1^,rpl20,,rpl21,rpl22,rpl23,rpl32,rpl33,rpl36,* | *rpl14,rpl16^1^,rpl21^1^,rpl20,rpl21,rpl22,rpl23,rpl32,rpl33,rpl36,* |
| Subunits of RNA polymerase | *rpoA,rpoB^3^,rpoC11^1^,rpoC2* | *rpoA,rpoB,rpoC1^1^,rpoC2* |
| Subunits of NADH dehydrogenase | *ndhA^1^,ndhC,ndhD,ndhE,ndhF^1^,ndhG,ndhH,ndhI,ndhJ,ndhK,* | *ndhA^1^,ndhB^2^,ndhC,ndhD,ndhE,ndhF,ndhG,ndhH,ndhI,ndhJ,ndhK,* |
| Subunits of Photosystem I | *psaA, B, C, I, J* | *psaA, B, C, I, J,M* |
| Subunits of Photosystem II | *psbA4^4^,psbB,psbC,psbD,psbE,psbF,psbH,psbI,psbJ,psbK,psbL,psbM,psbN,psbT,*  *psbZ* | *psbA,psbB,psbC,psbD,psbE,psbF,psbH,psbI,psbJ,psbK,psbL,psbM,psbN,psbT,psbZ* |
| Large subunit of RuBisco | *rbcL* | *rbcL* |
| Subunits of cytochrome b/f complex | *petA^1^,petB,petD,petG,petL,petN* | *petA,petB^a^,petD,petG,petL,petN* |
| Subunits of ATP synthase | *atpA^1^, B, E, F^1^, H, I* | *atpA, B, E, F^1^, H, I* |
| Acetyl-CoA carboxylase | *accD* | *accD* |
| C-type cytochrom synthesis gene | *ccsA* | *ccsA* |
| Maturase | *matK^1^* | *matK* |
| Protease | *clpP^2^* | *clpP^2^* |
| Envelope membrane protein | *cemA* | *cemA* |
| Conserved hypothetical cp reading frames | *ycf1^1^ , 2^4,6^, 3^1^, 4, 12* | *ycf12 , 2^2^, 3^2^, 4,* |
| Translational initiation factor | *infA^1^* | *infA* |
| protochlorophyllide reductase | *chlB^1^,chlL,chlN* | *chlB,chlL,chlN* |

^1^ Gene containing a single intron, ^2^ Gene containing two introns, ^3^ Gene containing three intron, ^4^ Two gene copies in the IRs, ^5^ Four gene copies in the IRs. ^6^ Gene containing four introns
