# Supplementary material for: Plastid genome and composition analysis of two medical ferns: Dryopteris crassirhizoma Nakai and Osmunda japonica Thunb
Source: Chin Med. 2019 Mar 14;14:9. doi: 10.1186/s13020-019-0230-4 (PMC6417082; doi:10.1186/s13020-019-0230-4)
Supplement: Supplementary file 3 — Additional file 3: Table S2. The SSR characteristic in Dryopteris crassirhizoma Nakai. [file 13020_2019_230_MOESM3_ESM.doc]

| **SSR nr.**  **Table S2** **The SSR characteristic in *Dryopteris crassirhizoma* Nakai plastid genome** | **SSR type** | **SSR** | **size** | **start** | **end** |
| --- | --- | --- | --- | --- | --- |
| 1 | p1 | (A)8 | 8 | 861 | 868 |
| 2 | p1 | (C)24 | 24 | 3862 | 3885 |
| 3 | p1 | (A)10 | 10 | 4051 | 4060 |
| 4 | p2 | (TA)8 | 16 | 4294 | 4309 |
| 5 | c | (T)8catgtcggtccccctccccacttccctttccatat(A)8 | 51 | 8148 | 8198 |
| 6 | c | (A)12ttaaataaatatatctgtttaattttttacaaaatttcttagtatca(AT)5 | 69 | 8450 | 8518 |
| 7 | c | (AT)6acaaaatatatacggaaacgaggagtgggt(G)10 | 52 | 8685 | 8736 |
| 8 | p1 | (G)16 | 16 | 10062 | 10077 |
| 9 | p1 | (A)8 | 8 | 12374 | 12381 |
| 10 | c | (T)8atacttcat(A)13 | 30 | 14071 | 14100 |
| 11 | p1 | (C)11 | 11 | 22237 | 22247 |
| 12 | p1 | (A)9 | 9 | 22650 | 22658 |
| 13 | p1 | (C)8 | 8 | 30130 | 30137 |
| 14 | c | (ATCT)3attcga(ATAG)4 | 34 | 31576 | 31609 |
| 15 | p1 | (T)11 | 11 | 31730 | 31740 |
| 16 | p1 | (T)10 | 10 | 34451 | 34460 |
| 17 | p2 | (TG)5 | 10 | 34677 | 34686 |
| 18 | p2 | (AT)10 | 20 | 35055 | 35074 |
| 19 | c | (CTAT)3(AGAT)3 | 24 | 35706 | 35729 |
| 20 | p1 | (T)10 | 10 | 36460 | 36469 |
| 21 | p1 | (C)10 | 10 | 41545 | 41554 |
| 22 | p1 | (A)9 | 9 | 43514 | 43522 |
| 23 | p1 | (A)12 | 12 | 45115 | 45126 |
| 24 | p1 | (A)8 | 8 | 45321 | 45328 |
| 25 | p1 | (G)8 | 8 | 49058 | 49065 |
| 26 | c | (TA)7tctc(AT)7 | 32 | 49637 | 49668 |
| 27 | p1 | (G)8 | 8 | 52082 | 52089 |
| 28 | c | (A)8ttgaatttgttccgaa(T)9ccacttctgaatcggccacatctta(C)13 | 71 | 55436 | 55506 |
| 29 | c | (A)10ttcga(G)10 | 25 | 56929 | 56953 |
| 30 | p3 | (TCC)4 | 12 | 60371 | 60382 |
| 31 | p1 | (A)8 | 8 | 61291 | 61298 |
| 32 | p4 | (TTCT)3 | 12 | 62424 | 62435 |
| 33 | p1 | (A)8 | 8 | 63972 | 63979 |
| 34 | p1 | (A)8 | 8 | 64273 | 64280 |
| 35 | p1 | (C)10 | 10 | 67263 | 67272 |
| 36 | c | (C)8ttctttttctt(G)15 | 34 | 71506 | 71539 |
| 37 | p1 | (A)8 | 8 | 75888 | 75895 |
| 38 | p1 | (C)10 | 10 | 76197 | 76206 |
| 39 | p1 | (T)9 | 9 | 76353 | 76361 |
| 40 | p1 | (A)9 | 9 | 78554 | 78562 |
| 41 | p1 | (C)8 | 8 | 80757 | 80764 |
| 42 | c | (T)8atacttttagaattcgaactcgat(TTC)4 | 44 | 81988 | 82031 |
| 43 | p1 | (T)10 | 10 | 83659 | 83668 |
| 44 | p1 | (G)13 | 13 | 89279 | 89291 |
| 45 | p1 | (G)14 | 14 | 97687 | 97700 |
| 46 | p4 | (TTTA)3 | 12 | 101561 | 101572 |
| 47 | p1 | (G)12 | 12 | 106465 | 106476 |
| 48 | c | (G)11caaccgatcgaggtcgagatcaaccccacaagccccccacgatctgtatcgatcagtcaccaattagtacttcccatt(C)8 | 97 | 106924 | 107020 |
| 49 | p2 | (AT)10 | 20 | 111400 | 111419 |
| 50 | p2 | (AT)9 | 18 | 111597 | 111614 |
| 51 | p1 | (C)15 | 15 | 115168 | 115182 |
| 52 | p1 | (C)8 | 8 | 115458 | 115465 |
| 53 | p4 | (ATAA)3 | 12 | 117284 | 117295 |
| 54 | p1 | (G)8 | 8 | 123766 | 123773 |
| 55 | c | (G)8aatgggaagtactaattggtgactgatcgatacagatcgtggggggcttgtggggttgatctcgacctcgatcggttg(C)11 | 97 | 129035 | 129131 |
| 56 | p1 | (C)12 | 12 | 129579 | 129590 |
| 57 | p4 | (ATAA)3 | 12 | 134482 | 134493 |
| 58 | p1 | (C)14 | 14 | 138355 | 138368 |
| 59 | p1 | (C)13 | 13 | 146764 | 146776 |
| 60 | p1 | (A)10 | 10 | 152387 | 152396 |
