# Supplementary material for: Plastid genome and composition analysis of two medical ferns: Dryopteris crassirhizoma Nakai and Osmunda japonica Thunb
Source: Chin Med. 2019 Mar 14;14:9. doi: 10.1186/s13020-019-0230-4 (PMC6417082; doi:10.1186/s13020-019-0230-4)
Supplement: Supplementary file 4 — Additional file 4: Table S3. The SSR characteristic in Osmunda japonica Thunb.. [file 13020_2019_230_MOESM4_ESM.doc]

**Table S3** **The SSR characteristic in *Osmunda japonica* Thunb. plastid genome**

| **SSR nr.** | **SSR type** | **SSR** | **size** | **start** | **end** |
| --- | --- | --- | --- | --- | --- |
| 1 | p1 | (T)9 | 9 | 1131 | 1139 |
| 2 | p1 | (C)11 | 11 | 2026 | 2036 |
| 3 | p1 | (A)11 | 11 | 2147 | 2157 |
| 4 | p3 | (ATT)4 | 12 | 2276 | 2287 |
| 5 | p1 | (A)9 | 9 | 5227 | 5235 |
| 6 | p1 | (T)8 | 8 | 5414 | 5421 |
| 7 | p1 | (T)9 | 9 | 5676 | 5684 |
| 8 | p4 | (TTAG)3 | 12 | 6084 | 6095 |
| 9 | p1 | (A)9 | 9 | 6561 | 6569 |
| 10 | p4 | (ATGA)3 | 12 | 9909 | 9920 |
| 11 | p2 | (TA)5 | 10 | 12008 | 12017 |
| 12 | p1 | (G)10 | 10 | 15285 | 15294 |
| 13 | c | (AGAA)3agagatatatcttgaagagattatag(A)9 | 47 | 19534 | 19580 |
| 14 | p2 | (TC)5 | 10 | 22566 | 22575 |
| 15 | p1 | (C)9 | 9 | 23663 | 23671 |
| 16 | p1 | (A)9 | 9 | 24008 | 24016 |
| 17 | p1 | (T)8 | 8 | 24478 | 24485 |
| 18 | p1 | (T)12 | 12 | 29294 | 29305 |
| 19 | p1 | (G)9 | 9 | 29539 | 29547 |
| 20 | c | (T)9ccccttttcccttcttctt(C)9 | 37 | 29904 | 29940 |
| 21 | p2 | (TC)5 | 10 | 42422 | 42431 |
| 22 | p1 | (C)8 | 8 | 43912 | 43919 |
| 23 | p1 | (A)8 | 8 | 44528 | 44535 |
| 24 | p3 | (TAT)4 | 12 | 44850 | 44861 |
| 25 | c | (T)15cctcctcttcctcct(C)8ataggaaggacctgcagtacgaatcattaatgtgagggaatatgagagggatttac(G)18 | 112 | 46193 | 46304 |
| 26 | c | (TA)7(T)8 | 22 | 47939 | 47960 |
| 27 | c | (TA)8(T)9 | 25 | 52616 | 52640 |
| 28 | p2 | (TC)5 | 10 | 53824 | 53833 |
| 29 | p2 | (TC)5 | 10 | 60611 | 60620 |
| 30 | p3 | (TAT)4 | 12 | 61068 | 61079 |
| 31 | p1 | (T)8 | 8 | 62129 | 62136 |
| 32 | p2 | (AT)13 | 26 | 63327 | 63352 |
| 33 | c | (TACTAT)3tactaatagtacttgtacttgtatgattagctaac(ATCT)3 | 65 | 65076 | 65140 |
| 34 | p1 | (A)9 | 9 | 66884 | 66892 |
| 35 | p2 | (TA)6 | 12 | 69845 | 69856 |
| 36 | p1 | (T)9 | 9 | 71828 | 71836 |
| 37 | p2 | (GA)5 | 10 | 72239 | 72248 |
| 38 | p4 | (AATG)3 | 12 | 76404 | 76415 |
| 39 | p1 | (T)10 | 10 | 81065 | 81074 |
| 40 | p3 | (TAA)4 | 12 | 81387 | 81398 |
| 41 | p1 | (A)14 | 14 | 82066 | 82079 |
| 42 | p1 | (A)8 | 8 | 82985 | 82992 |
| 43 | p1 | (C)17 | 17 | 84256 | 84272 |
| 44 | c* | (AT)8(T)9* | 24 | 84856 | 84879 |
| 45 | p1 | (T)9 | 9 | 85510 | 85518 |
| 46 | p4 | (GTTG)3 | 12 | 87616 | 87627 |
| 47 | p1 | (G)10 | 10 | 91900 | 91909 |
| 48 | p4 | (ATCA)3 | 12 | 93821 | 93832 |
| 49 | p1 | (A)8 | 8 | 94793 | 94800 |
| 50 | c | (T)11c(T)13caaaaaagtaaaaaaga(AT)9 | 60 | 95377 | 95436 |
| 51 | p4 | (TTCT)3 | 12 | 98183 | 98194 |
| 52 | p4 | (GAAT)3 | 12 | 98324 | 98335 |
| 53 | p4 | (GGTT)3 | 12 | 101575 | 101586 |
| 54 | p1 | (G)8 | 8 | 104187 | 104194 |
| 55 | p4 | (AGGT)3 | 12 | 107386 | 107397 |
| 56 | p1 | (A)10 | 10 | 109128 | 109137 |
| 57 | c | (G)11cgatcaaccatgaccaggatcttccgcaccag(C)9 | 52 | 110490 | 110541 |
| 58 | p4 | (ATTC)3 | 12 | 119268 | 119279 |
| 59 | c | (T)12caatcattcccccttctatacttccctatactcccccaggggaggtcctataccttcccttccc(G)9 | 85 | 120553 | 120637 |
| 60 | p1 | (T)8 | 8 | 122759 | 122766 |
| 61 | c | (A)12taa(AT)7 | 29 | 124596 | 124624 |
| 62 | p1 | (A)9 | 9 | 130421 | 130429 |
| 63 | p2 | (TC)5 | 10 | 130996 | 131005 |
| 64 | c | (G)9ctggtgcggaagatcctggtcatggttgatcg(C)11 | 52 | 133145 | 133196 |
| 65 | p1 | (T)10 | 10 | 134549 | 134558 |
| 66 | p4 | (CTAC)3 | 12 | 136287 | 136298 |
| 67 | p1 | (C)8 | 8 | 139492 | 139499 |
| 68 | p4 | (AACC)3 | 12 | 142100 | 142111 |
