# Supplementary material for: Plastid genome and composition analysis of two medical ferns: Dryopteris crassirhizoma Nakai and Osmunda japonica Thunb
Source: Chin Med. 2019 Mar 14;14:9. doi: 10.1186/s13020-019-0230-4 (PMC6417082; doi:10.1186/s13020-019-0230-4)
Supplement: Supplementary file 5 — Additional file 5: Table S4. Long repeat sequences in Osmunda japonica Thunb. plastid genome. [file 13020_2019_230_MOESM5_ESM.doc]

**Table S4 Long repeat sequences in *Osmunda japonca* Thunb. plastid genome**

| **ID** | **Length** | **Type** | **Repeat 1 start** | **Repeat 2 start** | **Mismatch (bp)** | **E-value** | **gene** | **region** |
| --- | --- | --- | --- | --- | --- | --- | --- | --- |
| R1 | 32 | F | 5830 | 6118 | 3 | 1.37E-03 | *_* | LSC |
| R2 | 46 | F | 5953 | 6032 | 3 | 1.56E-11 | *_* | LSC |
| R3 | 34 | F | 6043 | 6118 | 3 | 1.03E-04 | *_* | LSC |
| R4 | 31 | R | 18800 | 18800 | 2 | 6.70E-05 | *_* | LSC |
| R5 | 36 | P | 18809 | 18809 | 2 | 8.87E-08 | *_* | LSC |
| R6 | 31 | F | 23883 | 61228 | 3 | 4.95E-03 | *trnS-CGA,trnS-GCU* | LSC |
| R7 | 31 | P | 23887 | 24298 | 3 | 4.95E-03 | *trnS-CGA,trnS-GCU* | LSC |
| R8 | 30 | P | 24301 | 47718 | 1 | 1.79E-06 | *trnS-GCU,trnS-CGA* | LSC |
| R9 | 31 | P | 24303 | 61227 | 2 | 6.70E-05 | *trnS-GCU,trnS-GCU* | LSC |
| R10 | 31 | F | 47935 | 52614 | 3 | 4.95E-03 | *_* | LSC |
| R11 | 31 | F | 52615 | 84855 | 3 | 4.95E-03 | *_* | LSC |
| R12 | 30 | P | 52616 | 95406 | 3 | 1.79E-02 | *_* | LSC |
| R13 | 32 | F | 52616 | 95420 | 3 | 3.75E-04 | *_* | LSC |
| R14 | 32 | P | 52616 | 124594 | 3 | 1.37E-03 | *_* | LSC |
| R15 | 36 | F | 55402 | 57626 | 2 | 8.87E-08 | *psaB,psaA* | LSC |
| R16 | 33 | R | 63319 | 63327 | 3 | 3.75E-04 | *_* | LSC |
| R17 | 30 | P | 63325 | 63325 | 2 | 2.51E-04 | *_* | LSC |
| R18 | 34 | F | 63569 | 63602 | 3 | 2.09E-06 | *_* | LSC |
| R19 | 34 | F | 84847 | 95413 | 2 | 1.26E-06 | *_* | LSC |
| R20 | 34 | P | 84847 | 95410 | 2 | 1.26E-06 | *_* | LSC |
| R21 | 32 | P | 84852 | 124595 | 3 | 1.37E-03 | *_* | LSC |
| R22 | 46 | P | 95405 | 95405 | 0 | 1.17E-18 | *_* | LSC |
| R23 | 32 | F | 113601 | 113610 | 2 | 1.79E-05 | *_* | SSC |
| R24 | 34 | F | 124596 | 124599 | 3 | 2.82E-05 | *_* | SSC |
| R25 | 34 | F | 124596 | 124600 | 3 | 1.03E-04 | *_* | SSC |
| R26 | 32 | P | 124602 | 124602 | 2 | 1.79E-05 | *_* | SSC |

F forward, P palindromic, R reverse, - intergenic space
