# Supplementary material for: Plastid genome and composition analysis of two medical ferns: Dryopteris crassirhizoma Nakai and Osmunda japonica Thunb
Source: Chin Med. 2019 Mar 14;14:9. doi: 10.1186/s13020-019-0230-4 (PMC6417082; doi:10.1186/s13020-019-0230-4)
Supplement: Supplementary file 6 — Additional file 6: Table S5. Long repeat sequences in Dryopteris crassirhizoma Nakai plastid genome. [file 13020_2019_230_MOESM6_ESM.doc]

**Table S5 Long repeat sequences in *Dryopteris crassirhizoma* Nakai plastid genome**

| **ID** | **Length** | **Type** | **Repeat 2 start** | **Repeat 1 start** | **Mismatch(bp)** | **E-value** | **gene** | **region** |
| --- | --- | --- | --- | --- | --- | --- | --- | --- |
| R1 | 33 | R | 3857 | 3857 | 2 | 5.47E-06 | *_* | LSC |
| R2 | 32 | P | 7892 | 31417 | 2 | 5.47E-06 | *trnS-CGA,trnS-GCU* | LSC |
| R3 | 31 | P | 7896 | 43760 | 3 | 5.69E-03 | *trnS-CGA,trnS-GCU* | LSC |
| R4 | 30 | P | 7960 | 31350 | 3 | 2.06E-02 | *trnS-CGA,trnS-GCU* | LSC |
| R5 | 31 | P | 20519 | 20522 | 3 | 5.69E-03 | *_* | LSC |
| R6 | 32 | P | 31576 | 31576 | 0 | 3.60E-10 | *_* | LSC |
| R7 | 34 | F | 35033 | 35041 | 2 | 1.45E-06 | *_* | LSC |
| R8 | 34 | R | 35034 | 35047 | 3 | 1.18E-04 | *_* | LSC |
| R9 | 30 | C | 35040 | 49638 | 2 | 7.70E-05 | *_* | LSC |
| R10 | 30 | P | 35040 | 49637 | 2 | 7.70E-05 | *_* | LSC |
| R11 | 31 | P | 35041 | 49637 | 3 | 1.57E-03 | *_* | LSC |
| R12 | 33 | R | 35042 | 35048 | 3 | 4.32E-04 | *_* | LSC |
| R13 | 31 | R | 35050 | 35050 | 2 | 7.70E-05 | *_* | LSC |
| R14 | 30 | F | 36929 | 39144 | 3 | 5.69E-03 | *psaB,psaA* | LSC |
| R15 | 35 | F | 38112 | 40336 | 3 | 3.24E-05 | *psaB,psaA* | LSC |
| R16 | 36 | F | 42626 | 139998 | 2 | 1.02E-07 | *_* | LSC,IRb |
| R17 | 36 | P | 42626 | 96022 | 2 | 1.02E-07 | *_* | LSC,IRa |
| R18 | 34 | F | 46401 | 143436 | 3 | 1.18E-04 | *trnF-GAA,_* | LSC,IRb |
| R19 | 34 | P | 46401 | 92586 | 3 | 1.18E-04 | *trnF-GAA,_* | LSC,IRa |
| R20 | 30 | F | 49637 | 49639 | 3 | 2.06E-02 | *_* | LSC |
| R21 | 32 | R | 49637 | 49637 | 3 | 1.57E-03 | *_* | LSC |
| R22 | 32 | R | 49637 | 49637 | 2 | 2.05E-05 | *_* | LSC |
| R23 | 31 | P | 49638 | 49638 | 3 | 5.69E-03 | *_* | LSC |
| R24 | 30 | R | 49639 | 49639 | 2 | 2.88E-04 | *_* | LSC |
| R25 | 31 | P | 54251 | 63891 | 3 | 5.69E-03 | *_,rpl33* | LSC |
| R26 | 38 | F | 63107 | 63126 | 2 | 7.11E-09 | *_* | LSC |
| R27 | 31 | F | 74135 | 74187 | 3 | 5.69E-03 | *_,rpoA* | LSC |
| R28 | 44 | F | 74143 | 74169 | 3 | 2.50E-10 | *_,rpoA* | LSC |
| R29 | 31 | F | 84076 | 85109 | 3 | 5.69E-03 | *_,trnR-ACG* | IRa |
| R30 | 31 | P | 84076 | 150916 | 3 | 5.69E-03 | *_,trnR-ACG* | IRa,IRb |
| R31 | 31 | P | 85109 | 151949 | 3 | 5.69E-03 | *trnR-ACG* | IRa,IRb |
| R32 | 86 | F | 90688 | 92747 | 0 | 1.11E-42 | *rrn16* | IRa |
| R33 | 86 | P | 90688 | 143223 | 0 | 1.11E-42 | *rrn16* | IRa,IRb |
| R34 | 32 | F | 91873 | 93926 | 0 | 3.60E-10 | *rrn16* | IRa |
| R35 | 32 | P | 91873 | 142098 | 0 | 3.60E-10 | *rrn16* | IRa,IRb |
| R36 | 130 | F | 91905 | 93965 | 3 | 1.13E-60 | *rrn16* | IRa |
| R37 | 130 | P | 91905 | 141961 | 3 | 1.13E-60 | *rrn16* | IRa,IRb |
| R38 | 145 | F | 91984 | 94044 | 3 | 1.46E-69 | *rrn16* | IRa |
| R39 | 145 | P | 91984 | 141867 | 3 | 1.46E-69 | *rrn16* | IRa,IRb |
| R40 | 93 | F | 92036 | 94096 | 1 | 7.49E-44 | *rrn16* | IRa |
| R41 | 93 | P | 92036 | 141867 | 1 | 7.49E-44 | *rrn16* | IRa,IRb |
| R42 | 41 | F | 92088 | 94148 | 0 | 1.37E-15 | *rrn16* | IRa |
| R43 | 41 | P | 92088 | 141867 | 0 | 1.37E-15 | *rrn16* | IRa,IRb |
| R44 | 43 | F | 92129 | 94184 | 1 | 4.39E-14 | *_* | IRa |
| R45 | 43 | P | 92129 | 141829 | 1 | 4.39E-14 | *_* | IRa,IRb |
| R46 | 30 | F | 92151 | 94206 | 3 | 2.06E-02 | *_* | IRa |
| R47 | 30 | P | 92151 | 141820 | 3 | 2.06E-02 | *_* | IRa,IRb |
| R48 | 44 | F | 92182 | 94237 | 3 | 2.50E-10 | *_* | IRa |
| R49 | 44 | P | 92182 | 141775 | 3 | 2.50E-10 | *_* | IRa,IRb |
| R50 | 41 | F | 92191 | 94246 | 3 | 1.29E-08 | *_* | IRa |
| R51 | 41 | P | 92191 | 141769 | 3 | 1.29E-08 | *_* | IRa,IRb |
| R52 | 74 | F | 92271 | 94346 | 3 | 2.76E-28 | *_* | IRa |
| R53 | 74 | P | 92271 | 141635 | 3 | 2.76E-28 | *_* | IRa,IRb |
| R54 | 57 | F | 92288 | 94363 | 2 | 1.52E-20 | *_* | IRa |
| R55 | 57 | P | 92288 | 141635 | 2 | 1.52E-20 | *_* | IRa,IRb |
| R56 | 49 | F | 92349 | 94430 | 2 | 2.84E-15 | *_* | IRa |
| R57 | 49 | P | 92349 | 141577 | 2 | 2.84E-15 | *_* | IRa,IRb |
| R58 | 33 | F | 92365 | 94446 | 0 | 8.99E-11 | *_* | IRa |
| R59 | 33 | P | 92365 | 141577 | 0 | 8.99E-11 | *_* | IRa,IRb |
| R60 | 32 | F | 92449 | 94509 | 3 | 1.57E-03 | *_* | IRa |
| R61 | 32 | P | 92449 | 141516 | 3 | 1.57E-03 | *_* | IRa,IRb |
| R62 | 61 | F | 92472 | 94540 | 2 | 2.63E-22 | *_* | IRa |
| R63 | 61 | P | 92472 | 141455 | 2 | 2.63E-22 | *_* | IRa,IRb |
| R64 | 45 | F | 92501 | 94569 | 3 | 6.69E-11 | *_* | IRa |
| R65 | 45 | P | 92501 | 141442 | 3 | 6.69E-11 | *_* | IRa,IRb |
| R66 | 35 | F | 92515 | 94583 | 3 | 3.24E-05 | *_* | IRa |
| R67 | 35 | P | 92515 | 141438 | 3 | 3.24E-05 | *_* | IRa,IRb |
| R68 | 86 | P | 92747 | 145282 | 0 | 1.11E-42 | *rrn16* | IRa,IRb |
| R69 | 32 | P | 93926 | 144151 | 0 | 3.60E-10 | *rrn16* | IRa,IRb |
| R70 | 130 | P | 93965 | 144021 | 3 | 1.13E-60 | *rrn16* | IRa,IRb |
| R71 | 145 | P | 94044 | 143927 | 3 | 1.46E-69 | *rrn16* | IRa,IRb |
| R72 | 93 | P | 94096 | 143927 | 1 | 7.49E-44 | *rrn16* | IRa,IRb |
| R73 | 41 | P | 94148 | 143927 | 0 | 1.37E-15 | *rrn16* | IRa,IRb |
| R74 | 43 | P | 94184 | 143884 | 1 | 4.39E-14 | *_* | IRa,IRb |
| R75 | 30 | P | 94206 | 143875 | 3 | 2.06E-02 | *_* | IRa,IRb |
| R76 | 44 | P | 94237 | 143830 | 3 | 2.50E-10 | *_* | IRa,IRb |
| R77 | 41 | P | 94246 | 143824 | 3 | 1.29E-08 | *_* | IRa,IRb |
| R78 | 75 | P | 94346 | 143711 | 3 | 2.76E-28 | *_* | IRa,IRb |
| R79 | 58 | P | 94363 | 143711 | 2 | 1.52E-20 | *_* | IRa,IRb |
| R80 | 49 | P | 94430 | 143658 | 2 | 2.84E-15 | *_* | IRa,IRb |
| R81 | 33 | P | 94446 | 143658 | 0 | 8.99E-11 | *_* | IRa,IRb |
| R82 | 32 | P | 94509 | 143575 | 3 | 1.57E-03 | *_* | IRa,IRb |
| R83 | 61 | P | 94540 | 143523 | 2 | 2.63E-22 | *_* | IRa,IRb |
| R84 | 45 | P | 94569 | 143510 | 3 | 6.69E-11 | *_* | IRa,IRb |
| R85 | 35 | P | 94583 | 143506 | 3 | 3.24E-05 | *_* | IRa,IRb |
| R86 | 38 | F | 105628 | 105652 | 2 | 7.11E-09 | *ycf2-D2* | IRa |
| R87 | 38 | P | 105628 | 130366 | 2 | 7.11E-09 | *ycf2-D2,ycf2* | IRa,IRb |
| R88 | 50 | F | 105628 | 105640 | 2 | 7.38E-16 | *ycf2-D2* | IRa |
| R89 | 50 | P | 105628 | 130366 | 2 | 7.38E-16 | *ycf2-D2,ycf2* | IRa,IRb |
| R90 | 50 | P | 105640 | 130378 | 2 | 7.38E-16 | *ycf2-D2,ycf2* | IRa,IRb |
| R91 | 38 | P | 105652 | 130390 | 2 | 7.11E-09 | *ycf2-D2,ycf2* | IRa,IRb |
| R92 | 38 | F | 130366 | 130390 | 2 | 7.11E-09 | *ycf2* | IRb |
| R93 | 50 | F | 130366 | 130378 | 2 | 7.38E-16 | *ycf2* | IRb |
| R94 | 30 | F | 130374 | 130398 | 1 | 2.06E-06 | *ycf2* | IRb |
| R95 | 42 | F | 130374 | 130386 | 1 | 1.72E-13 | *ycf2* | IRb |
| R96 | 45 | F | 141442 | 143510 | 3 | 6.69E-11 | *_* | IRb |
| R97 | 61 | F | 141455 | 143523 | 2 | 2.63E-22 | *_* | IRb |
| R98 | 42 | F | 141474 | 143542 | 1 | 1.72E-13 | *_* | IRb |
| R99 | 31 | F | 141488 | 143556 | 2 | 7.70E-05 | *_* | IRb |
| R100 | 32 | F | 141518 | 143578 | 3 | 1.57E-03 | *_* | IRb |
| R101 | 49 | F | 141577 | 143658 | 2 | 2.84E-15 | *_* | IRb |
| R102 | 75 | F | 141635 | 143711 | 3 | 2.76E-28 | *_* | IRb |
| R103 | 44 | F | 141775 | 143830 | 3 | 2.50E-10 | *_* | IRb |
| R104 | 36 | F | 141788 | 143843 | 3 | 8.83E-06 | *_* | IRb |
| R105 | 43 | F | 141829 | 143884 | 1 | 4.39E-14 | *_* | IRb |
| R106 | 145 | F | 141867 | 143927 | 3 | 1.46E-69 | *rrn16* | IRb |
| R107 | 130 | F | 141961 | 144021 | 3 | 1.13E-60 | *rrn16* | IRb |
| R108 | 84 | F | 142007 | 144067 | 2 | 7.12E-36 | *rrn16* | IRb |
| R109 | 59 | F | 143164 | 145217 | 0 | 2.00E-26 | *rrn16* | IRb |
| R110 | 72 | F | 143310 | 145369 | 0 | 2.97E-34 | *rrn16* | IRb |
| R111 | 31 | F | 150916 | 151949 | 3 | 5.69E-03 | *trnR-ACG* | IRb |

F forward, P palindromic, R reverse, - intergenic space
