# Supplementary material for: Plastid genome and composition analysis of two medical ferns: Dryopteris crassirhizoma Nakai and Osmunda japonica Thunb
Source: Chin Med. 2019 Mar 14;14:9. doi: 10.1186/s13020-019-0230-4 (PMC6417082; doi:10.1186/s13020-019-0230-4)
Supplement: Supplementary file 7 — Additional file 7: Table S6. The Pi value of coding regions and no-coding regions of Dryopteris. [file 13020_2019_230_MOESM7_ESM.doc]

**Table S6 The Pi value of coding region and no-coding region of *Dryopteris* plastid genomes**

| **Coding region** | | | **No-coding region** | | |
| --- | --- | --- | --- | --- | --- |
| **Gene** | **Length** | **Pi** | **Gene** | **Length** | **Pi** |
| *matK-CDS1* | 1239 | 0.0616088 | *start-matK* | 1467 | 0.0719274 |
| *matK-CDS2* | 252 | 0.037037 | *matK-rps16* | 721 | 0.0706767 |
| *rps16-CDS1* | 213 | 0.0500782 | *rps16-rps16* | 837 | 0.08895 |
| *rps16-CDS2* | 45 | 0.0148148 | *rps16-chlB* | 173 | 0.06621 |
| *chlB-CDS1* | 1161 | 0.0298593 | *chlB-chlB* | 137 | 0.0197531 |
| *chlB-CDS2* | 249 | 0.0160643 | *chlB-trnQ-UUG* | 232 | 0.0625 |
| *psbK* | 177 | 0.2022222 | *trnQ-UUG-psbK* | 392 | 0.0744986 |
| *psbI* | 111 | 0.024024 | *trnS-CGA-ycf12* | 577 | 0.0924574 |
| *ycf12* | 102 | 0.0392157 | *ycf12-trnR-UCU* | 1294 | 0.0649829 |
| *atpA-CDS1* | 495 | 0.0282828 | *trnR-UCU-atpA* | 138 | 0.0818713 |
| *atpA-CDS2* | 900 | 0.0333704 | *atpA-atpF* | 222 | 0.033033 |
| *atpF-CDS1* | 435 | 0.035249 | *atpF-atpF* | 712 | 0.0334288 |
| *atpF-CDS2* | 147 | 0.0362812 | *atpF-atpH* | 357 | 0.0713587 |
| *atpH* | 234 | 0.02849 | *atpI-rps2* | 597 | 0.0475362 |
| *atpI* | 660 | 0.0365297 | *rps2-rpoC2* | 129 | 0.0416667 |
| *rps2* | 396 | 0.035533 | *rpoC2-rpoC1* | 476 | 0.0299786 |
| *rpoC2* | 3984 | 0.0482886 | *rpoC1-rpoC1* | 676 | 0.0357143 |
| *rpoC1-CDS1* | 1642 | 0.0313582 | *rpoC1-rpoB* | 229 | 0.0380117 |
| *rpoC1-CDS2* | 257 | 0.0311284 | *rpoB-rpoB* | 155 | 0.01766 |
| *rpoB-CDS1* | 633 | 0.0242233 | *rpoB-trnD-GUC* | 1041 | 0.0721471 |
| *rpoB-CDS2* | 672 | 0.0473313 | *trnE-UUC-psbM* | 440 | 0.0560821 |
| *rpoB-CDS3* | 927 | 0.0237325 | *psbM-petN* | 1303 | 0.0819351 |
| *rpoB-CDS4* | 423 | 0.0378251 | *petN-trnC-GCA* | 451 | 0.0668185 |
| *psbM* | 105 | 0.0326797 | *trnC-GCA-trnG-UCC* | 519 | 0.0973085 |
| *petN* | 99 | 0.040404 | *trnG-UCC-psbZ* | 250 | 0.0755735 |
| *psbZ* | 189 | 0.0324324 | *psbZ-trnS-GCU* | 392 | 0.0501792 |
| *psbC* | 1296 | 0.0262346 | *trnS-GCU-psbC* | 313 | 0.0953947 |
| *psbD* | 1062 | 0.0194601 | *psbD-trnT-UGU* | 1350 | 0.0643697 |
| *psaB* | 2208 | 0.0220411 | *trnT-UGU-trnfM-CAU* | 495 | 0.0888889 |
| *psaA* | 2253 | 0.0266312 | *trnfM-CAU-psaB* | 696 | 0.040404 |
| *ycf3-CDS1* | 340 | 0.0372549 | *psaA-ycf3* | 972 | 0.0373832 |
| *ycf3-CDS2* | 146 | 0.0183908 | *ycf3-ycf3* | 769 | 0.0421607 |
| *rps4* | 588 | 0.0362812 | *ycf3-trnS-GCU* | 260 | 0.0535475 |
| *ndhJ* | 513 | 0.0272904 | *trnS-GCU-rps4* | 439 | 0.0764381 |
| *ndhK* | 738 | 0.0375396 | *rps4-trnL-UAA* | 659 | 0.061651 |
| *ndhC* | 417 | 0.0223821 | *trnL-UAA-trnL-UAA* | 623 | 0.0556492 |
| *atpE* | 399 | 0.0233918 | *trnL-UAA-trnF-GAA* | 279 | 0.0561661 |
| *atpB* | 1482 | 0.0238417 | *trnF-GAA-ndhJ* | 219 | 0.0952381 |
| *rbcL* | 1428 | 0.0294118 | *ndhC-trnV-UAC* | 502 | 0.085595 |
| *accD* | 939 | 0.0312389 | *trnV-UAC-trnV-UAC* | 648 | 0.088172 |
| *psaI* | 111 | 0.024024 | *trnM-CAU-atpE* | 153 | 0.086758 |
| *ycf4* | 555 | 0.039783 | *atpB-rbcL* | 644 | 0.0479167 |
| *cemA* | 1500 | 0.0346667 | *rbcL-accD* | 533 | 0.0777565 |
| *petA-CDS1* | 276 | 0.0217391 | *accD-psaI* | 353 | 0.1028684 |
| *petA-CDS2* | 684 | 0.0341131 | *psaI-ycf4* | 422 | 0.0837398 |
| *psbJ* | 126 | 0.016 | *ycf4-cemA* | 184 | 0.0604396 |
| *psbL* | 117 | 0.0405797 | *cemA-petA* | 206 | 0.038835 |
| *psbF* | 120 | 0.0277778 | *petA-psbJ* | 774 | 0.088763 |
| *psbE* | 252 | 0.0291005 | *psbJ-psbL* | 119 | 0.0282486 |
| *petL* | 96 | 0.0347222 | *psbE-petL* | 773 | 0.0501119 |
| *petG* | 117 | 0 | *petL-petG* | 151 | 0.0781609 |
| *psaJ* | 129 | 0.0361757 | *petG-trnW-CCA* | 257 | 0.0625 |
| *rpl33* | 207 | 0.041868 | *trnW-CCA-trnP-UGG* | 194 | 0.0891266 |
| *rps18* | 271 | 0.0345679 | *trnP-UGG-psaJ* | 395 | 0.0961366 |
| *rpl20* | 357 | 0.0280112 | *rps18-rpl20* | 242 | 0.0944206 |
| *clpP-CDS1* | 281 | 0.0260973 | *clpP-CDS1-clpP-CDS2* | 551 | 0.0449083 |
| *clpP-CDS2* | 292 | 0.0205479 | *clpP-CDS2-clpP-CDS3* | 693 | 0.0541605 |
| *clpP-CDS3* | 66 | 0.020202 | *clpP-CDS3-psbB* | 401 | 0.0641667 |
| *psbB* | 1614 | 0.031392 | *psbB-psbT* | 209 | 0.0464345 |
| *psbT* | 120 | 0.0333333 | *psbT-psbN* | 134 | 0.0547264 |
| *psbN* | 138 | 0.0193237 | *psbH-petB* | 968 | 0.1123321 |
| *psbH* | 225 | 0.038864 | *petB-petD* | 781 | 0.044182 |
| *petB* | 663 | 0.029382 | *petD-rpoA* | 391 | 0.0751174 |
| *petD* | 531 | 0.0175769 | *rpl36-infA* | 137 | 0.0661765 |
| *rpoA* | 1002 | 0.0419162 | *infA-rps8* | 141 | 0.0821256 |
| *rps11-CDS1* | 270 | 0.0271605 | *rps8-rpl14* | 250 | 0.0566802 |
| *rps11-CDS2* | 120 | 0.0166667 | *rpl14-rpl16* | 187 | 0.1333333 |
| *rpl36* | 108 | 0.0185185 | *rpl16-rps3* | 813 | 0.0434783 |
| *infA-CDS1* | 120 | 0.0222222 | *rps3-rpl22* | 352 | 0.0573066 |
| *infA-CDS2* | 66 | 0.030303 | *rpl2-rpl2* | 798 | 0.064389 |
| *rps8* | 363 | 0.0330579 | *rpl2-rpl23* | 121 | 0.0936639 |
| *rpl14* | 429 | 0.0312989 | *rpl23-trnI-CAU* | 164 | 0.1022495 |
| *rpl16* | 489 | 0.0463531 | *rps12-rps7-D2* | 645 | 0.0138004 |
| *rps3* | 543 | 0.0208717 | *rps7-D2-psbA* | 483 | 0.0452937 |
| *rpl22* | 249 | 0.0809717 | *psbA-trnH-GUG* | 422 | 0.0290557 |
| *rps19* | 234 | 0.034188 | *ycf2-D2-ycf2-D2* | 218 | 0.0216049 |
| *rpl2-CDS1* | 375 | 0.0195556 | *ycf2-D2-trnN-GUU* | 689 | 0.0353535 |
| *rpl2-CDS2* | 354 | 0.0357815 | *trnN-GUU-ndhF* | 478 | 0.042735 |
| *rpl23* | 270 | 0.054321 | *ndhF-rpl21* | 320 | 0.0546793 |
| *rps12* | 315 | 0.010582 | *rpl21-rpl32* | 269 | 0.0597015 |
| *rps7-D2-CDS1* | 138 | 0.0194647 | *rpl32-trnP-GGG* | 631 | 0.112605 |
| *rps7-D2-CDS2* | 312 | 0.0021368 | *trnP-GGG-trnL-UAG* | 290 | 0.1025641 |
| *psbA* | 1062 | 0.0100439 | *trnL-UAG-ccsA* | 304 | 0.0812721 |
| *ycf2-D2-CDS1* | 195 | 0.0205128 | *ccsA-ndhD* | 353 | 0.0818882 |
| *ycf2-D2-CDS2* | 1146 | 0.0280702 | *ndhD-psaC* | 148 | 0.0724638 |
| *ycf2-D2-CDS3* | 2055 | 0.0217356 | *ndhI-ndhA* | 152 | 0.0821918 |
| *ycf2-D2-CDS4* | 894 | 0.0074571 | *ndhA-ndhA* | 925 | 0.0494905 |
| *ycf2-D2-CDS5* | 1734 | 0.0150987 | *ndhH-rps15* | 106 | 0.0571429 |
| *ndhF-CDS1* | 1572 | 0.0729234 | *rps15-ycf1* | 263 | 0.0887199 |
| *ndhF-CDS2* | 228 | 0.0350877 | *ycf1-chlN* | 375 | 0.0800377 |
| *ndhF-CDS3* | 354 | 0.039548 | *chlN-chlL* | 225 | 0.0418535 |
| *rpl21* | 273 | 0.029304 |  |  |  |
| *rpl32* | 177 | 0.0696798 |  |  |  |
| *ccsA* | 939 | 0.0651709 |  |  |  |
| *ndhD* | 1509 | 0.0357853 |  |  |  |
| *psaC* | 246 | 0.0189702 |  |  |  |
| *ndhE* | 303 | 0.0330033 |  |  |  |
| *ndhG* | 699 | 0.045214 |  |  |  |
| *ndhI* | 522 | 0.0319285 |  |  |  |
| *ndhA-CDS1* | 581 | 0.0345423 |  |  |  |
| *ndhA-CDS2* | 577 | 0.0310078 |  |  |  |
| *ndhH* | 1179 | 0.0305862 |  |  |  |
| *rps15* | 306 | 0.0372807 |  |  |  |
| *ycf1-CDS1* | 4125 | 0.057379 |  |  |  |
| *ycf1-CDS2* | 993 | 0.0543807 |  |  |  |
| *chlN* | 1386 | 0.0519481 |  |  |  |
| *chlL* | 696 | 0.044316 |  |  |  |
