# Supplementary material for: Plastid genome and composition analysis of two medical ferns: Dryopteris crassirhizoma Nakai and Osmunda japonica Thunb
Source: Chin Med. 2019 Mar 14;14:9. doi: 10.1186/s13020-019-0230-4 (PMC6417082; doi:10.1186/s13020-019-0230-4)
Supplement: Supplementary file 8 — Additional file 8: Table S7. The Pi value of coding region and no-coding region of Osmunda. [file 13020_2019_230_MOESM8_ESM.doc]

**Table S7 The Pi value of coding region and no-coding region of *Osmunda* plastid genomes**

| **Coding region** | | | **No-coding region** | | |
| --- | --- | --- | --- | --- | --- |
| **Gene** | **Length** | **Pi** | **Gene** | **Length** | **Pi** |
| *rps12-CDS1* | 315 | 0.031746 | *start-rps12* | 560 | 0.1703704 |
| *rps7* | 468 | 0.042735 | *rps12-rps7* | 525 | 0.0957031 |
| *ndhB-CDS1* | 723 | 0.0594744 | *rps7-ndhB* | 361 | 0.1636905 |
| *ndhB-CDS2* | 762 | 0.0472441 | *ndhB-ndhB* | 726 | 0.0984743 |
| *ycf2-CDS3* | 1590 | 0.0994152 | *ndhB-trnL-CAA* | 923 | 0.137457 |
| *psbA* | 1062 | 0.0310734 | *ycf2-trnH-GUG* | 291 | 0.1042471 |
| *matK* | 1576 | 0.0899809 | *psbA-trnK-UUU* | 476 | 0.152968 |
| *rps16-CDS1* | 213 | 0.0516432 | *trnK-UUU-matK* | 144 | 0.0769231 |
| *rps16-CDS2* | 42 | 0.0238095 | *matK-trnK-UUU* | 764 | 0.0910384 |
| *chlB* | 1542 | 0.0331169 | *trnK-UUU-rps16* | 583 | 0.1073944 |
| *psbK* | 177 | 0.0282486 | *rps16-rps16* | 772 | 0.0790155 |
| *psbI* | 111 | 0.018018 | *rps16-chlB* | 149 | 0.0816327 |
| *psaM* | 99 | 0.0505051 | *chlB-trnQ-UUG* | 185 | 0.1270718 |
| *ycf12* | 102 | 0.029703 | *trnQ-UUG-psbK* | 300 | 0.04 |
| *atpA* | 1524 | 0.0282709 | *psbK-trnS-CGA* | 388 | 0.1591512 |
| *atpF-CDS1* | 411 | 0.0364964 | *trnS-GCU-psaM* | 264 | 0.1361868 |
| *atpF-CDS2* | 159 | 0.0512821 | *psaM-ycf12* | 278 | 0.0763636 |
| *atpI* | 747 | 0.0362416 | *ycf12-trnR-UCU* | 1196 | 0.0860034 |
| *rps2* | 726 | 0.0399449 | *atpF-atpF* | 646 | 0.0729814 |
| *rpoC2* | 4239 | 0.0508274 | *atpF-atpH* | 241 | 0.0982906 |
| *rpoC1-CDS1* | 1626 | 0.0393604 | *atpH-atpI* | 677 | 0.0913313 |
| *rpoC1-CDS2* | 432 | 0.0277778 | *atpI-rps2* | 201 | 0.079602 |
| *rpoB* | 3213 | 0.037037 | *rps2-rpoC2* | 136 | 0.0909091 |
| *petN* | 90 | 0.0444444 | *rpoC2-rpoC1* | 133 | 0.0601504 |
| *psbM* | 105 | 0.0190476 | *rpoC1-rpoC1* | 644 | 0.066874 |
| *psbZ* | 189 | 0.010582 | *rpoB-trnC-GCA* | 308 | 0.1331058 |
| *psbC* | 1422 | 0.0344585 | *trnC-GCA-petN* | 405 | 0.1120219 |
| *psbD* | 1062 | 0.0301318 | *petN-psbM* | 1323 | 0.0749035 |
| *rps14* | 303 | 0.0429043 | *psbM-trnD-GUC* | 839 | 0.089372 |
| *psaB* | 2205 | 0.0272109 | *trnY-GUA-trnE-UUC* | 137 | 0.0902256 |
| *psaA* | 2253 | 0.0292943 | *trnE-UUC-trnG-UCC* | 683 | 0.1360124 |
| *ycf3-CDS1* | 206 | 0.0194175 | *trnG-UCC-psbZ* | 247 | 0.0938776 |
| *ycf3-CDS2* | 213 | 0.0331754 | *psbZ-trnS-CGA* | 357 | 0.0859599 |
| *ycf3-CDS3* | 124 | 0.0403226 | *trnS-CGA-psbC* | 791 | 0.1152263 |
| *rps4* | 621 | 0.0420032 | *psbD-trnT-GGU* | 1280 | 0.0951239 |
| *ndhJ* | 477 | 0.0377358 | *trnT-GGU-trnfM-CAU* | 1138 | 0.1248852 |
| *ndhK* | 777 | 0.0453956 | *trnfM-CAU-rps14* | 124 | 0.1048387 |
| *ndhC* | 363 | 0.0385675 | *rps14-psaB* | 206 | 0.0684211 |
| *atpE* | 399 | 0.037594 | *psaA-ycf3-CDS1* | 342 | 0.0722892 |
| *atpB* | 1482 | 0.0412441 | *ycf3-CDS1-ycf3-CDS2* | 622 | 0.0610932 |
| *rbcL* | 1428 | 0.0427171 | *ycf3-CDS2-ycf3-CDS3* | 693 | 0.0591631 |
| *accD* | 933 | 0.0439443 | *ycf3-CDS3-trnS-GCU* | 475 | 0.1233184 |
| *psaI* | 111 | 0.036036 | *trnS-GCU-rps4* | 424 | 0.1187335 |
| *ycf4* | 555 | 0.0576577 | *rps4-trnT-GGU* | 241 | 0.0954357 |
| *cemA* | 1521 | 0.069691 | *trnT-GGU-trnF-GAA* | 1505 | 0.1136364 |
| *petA* | 1004 | 0.045045 | *trnF-GAA-ndhJ* | 481 | 0.1101322 |
| *psbJ* | 123 | 0.0083333 | *ndhC-trnV-UAC* | 485 | 0.1652893 |
| *psbL* | 117 | 0.0173913 | *trnV-UAC-trnV-UAC* | 588 | 0.0814558 |
| *psbF* | 120 | 0.0166667 | *trnV-UAC-trnM-CAU* | 156 | 0.12 |
| *psbE* | 252 | 0.0396825 | *trnM-CAU-atpE* | 150 | 0.1 |
| *petL* | 108 | 0.0566038 | *atpB-rbcL* | 658 | 0.0754414 |
| *petG* | 117 | 0.042735 | *rbcL-trnR-CCG* | 120 | 0.0756303 |
| *psaJ* | 129 | 0.0465116 | *trnR-CCG-accD* | 354 | 0.1120944 |
| *rpl33* | 201 | 0.0547264 | *accD-psaI* | 436 | 0.0851582 |
| *rps18* | 228 | 0.0307018 | *psaI-ycf4* | 426 | 0.1124402 |
| *rpl20* | 348 | 0.0632184 | *ycf4-cemA* | 189 | 0.0944444 |
| *rps12-CDS2* | 114 | 0.0263158 | *cemA-petA* | 251 | 0.068 |
| *clpP-CDS1* | 255 | 0.0509804 | *petA-psbJ* | 1045 | 0.1165703 |
| *clpP-CDS2* | 291 | 0.0378007 | *psbJ-psbL* | 115 | 0.0727273 |
| *clpP-CDS3* | 69 | 0 | *psbE-petL* | 1084 | 0.1260745 |
| *psbB* | 1527 | 0.0340537 | *petL-petG* | 121 | 0.0330579 |
| *psbT* | 108 | 0.0462963 | *petG-trnW-CCA* | 150 | 0.0895522 |
| *psbN* | 132 | 0.0151515 | *trnW-CCA-trnP-UGG* | 203 | 0.1315789 |
| *psbH* | 225 | 0.059633 | *trnP-UGG-psaJ* | 226 | 0.081448 |
| *petB-CDS2* | 642 | 0.0342679 | *psaJ-rpl33* | 181 | 0.1162791 |
| *petD* | 550 | 0.0455373 | *rps18-rpl20* | 224 | 0.1474654 |
| *rpoA* | 1026 | 0.0545809 | *rpl20-rps12* | 956 | 0.0825991 |
| *rps11* | 393 | 0.0305344 | *rps12-clpP-CDS1* | 365 | 0.1264368 |
| *rpl36* | 114 | 0.0263158 | *clpP-CDS1-clpP-CDS2* | 535 | 0.1166008 |
| *infA* | 264 | 0.0492424 | *clpP-CDS2-clpP-CDS3* | 706 | 0.0725462 |
| *rps8* | 399 | 0.0701754 | *clpP-CDS3-psbB* | 443 | 0.1264368 |
| *rpl14* | 369 | 0.0653951 | *psbB-psbT* | 241 | 0.1166667 |
| *rpl16-CDS1* | 429 | 0.037296 | *psbT-psbN* | 100 | 0.1145833 |
| *rps3* | 657 | 0.047546 | *psbH-petB* | 132 | 0.1 |
| *rpl22* | 407 | 0.0606061 | *petB-petB* | 764 | 0.0959264 |
| *rps19* | 279 | 0.0322581 | *petB-petD* | 832 | 0.0842359 |
| *rpl2-CDS1* | 452 | 0.04 | *infA-rps8* | 121 | 0.0689655 |
| *rpl2-CDS2* | 397 | 0.0554156 | *rps8-rpl14* | 383 | 0.176 |
| *rpl23* | 273 | 0.043956 | *rpl14-rpl16* | 194 | 0.1637427 |
| *ndhF* | 2223 | 0.0702703 | *rpl16-rpl16* | 767 | 0.1012146 |
| *rpl21* | 357 | 0.047619 | *rpl16-rps3* | 142 | 0.1642857 |
| *rpl32* | 180 | 0.0277778 | *rpl2-rpl2* | 677 | 0.0950226 |
| *ccsA* | 966 | 0.0559006 | *rpl23-trnI-CAU* | 223 | 0.1302326 |
| *ndhD* | 1500 | 0.0433333 | *trnI-CAU-trnV-GAC* | 1235 | 0.0454545 |
| *psaC* | 246 | 0.0325203 | *trnV-GAC-rrn16* | 214 | 0.0246305 |
| *ndhE* | 303 | 0.0363036 | *rrn16-trnI-GAU* | 259 | 0.0501931 |
| *ndhG* | 597 | 0.0555556 | *trnI-GAU-trnI-GAU* | 934 | 0.0364416 |
| *ndhI* | 576 | 0.0572917 | *trnA-UGC-trnA-UGC* | 780 | 0.0142119 |
| *ndhA-CDS1* | 552 | 0.0491803 | *trnA-UGC-rrn23* | 152 | 0.0337838 |
| *ndhA-CDS2* | 558 | 0.0448029 | *rrn5-trnR-ACG* | 226 | 0.0410959 |
| *ndhH* | 1182 | 0.0279188 | *trnR-ACG-trnN-GUU* | 636 | 0.0343137 |
| *rps15* | 267 | 0.0636704 | *ndhF-rpl21* | 338 | 0.1097561 |
| *chlN* | 1401 | 0.0578158 | *rpl21-rpl32* | 178 | 0.0887574 |
| *chlL* | 879 | 0.0333333 | *rpl32-trnP-GGG* | 1784 | 0.1476868 |
|  |  |  | *trnP-GGG-trnL-UAG* | 132 | 0.1287879 |
|  |  |  | *ccsA-ndhD* | 290 | 0.1296296 |
|  |  |  | *ndhD-psaC* | 100 | 0.0736842 |
|  |  |  | *psaC-ndhE* | 397 | 0.0775401 |
|  |  |  | *ndhG-ndhI* | 267 | 0.151751 |
|  |  |  | *ndhA-ndhA* | 887 | 0.0813288 |
|  |  |  | *rps15-chlN* | 5563 | 0.0863661 |
